# Timing of Complementary Feeding in Preterm Infants and Prevalence of Overweight and Obesity: A Randomized Clinical Trial

**DOI:** 10.1001/jamanetworkopen.2025.2968

**Published:** 2025-04-30

**Authors:** Karin M. Vissers, Edith J. M. Feskens, Johannes B. van Goudoever, Arieke J. Janse

**Affiliations:** 1 Department of Pediatrics, Hospital Gelderse Vallei, Ede, the Netherlands; 2 Department of Pediatrics, Amsterdam University Medical Center, University of Amsterdam, Vrije Universiteit, Emma Children’s Hospital, Amsterdam, the Netherlands; 3 Division of Human Nutrition and Health, Wageningen University, Wageningen, the Netherlands

## Abstract

**Importance:**

The appropriate time for initiating complementary feeding in preterm infants is crucial for optimizing growth and preventing long-term health issues, such as overweight. Currently, there are no established guidelines for preterm infants.

**Objective:**

To investigate the effect of initiating complementary feeding at corrected age 12 weeks vs 17 weeks on the prevalence of overweight and obesity at corrected age 2 years in preterm infants.

**Design, Setting, and Participants:**

This multicenter randomized clinical trial was conducted between May 13, 2016, and April 26, 2021, with follow-up completed in December 2023. Seventeen hospitals in the Netherlands recruited preterm infants born between gestational age (GA) 30 and 36 weeks, supplemented with a reference group of full-term infants.

**Intervention:**

Preterm infants were randomized to initiating complementary feeding at corrected age 12 weeks (early group) or 17 weeks (late group).

**Main Outcomes and Measures:**

The primary outcome was the prevalence of overweight and obesity at corrected age 2 years measured using logistic mixed models. Secondary outcomes were height, weight, head circumference, body mass index, and *z* scores at corrected age 1 and 2 years, as well as neurodevelopment, atopic dermatitis score, and health-related quality of life.

**Results:**

A total of 255 preterm infants were included and randomly assigned, with 131 (51.4%; median [IQR] GA, 34 weeks 2 days [32 weeks 5 days to 35 weeks 1 day]; 77 male [58.8%]) allocated to the early group and 124 (48.6%; median [IQR] GA, 34 weeks 0 days [32 weeks 6 days to 34 weeks 6 days]; 62 male [50.0%]) allocated to the late group. A total of 159 full-term infants (median [IQR] GA, 40 weeks 0 days [39 week 0 days to 41 weeks 0 days]; 84 female [52.8%]) were included as the reference group. Information on the primary outcome was available for 226 preterm infants (88.6%) and 144 full-term infants (90.6%). At corrected age 2 years, the prevalence of overweight was 6.0% (95% CI, 2.7%-11.5%) in the early group and 5.5% (95% CI, 2.3%-11.1%) in the late group. For obesity, the prevalence was 1.7% (95% CI, 0.3%-5.5%) in the early group and 1.8% (95% CI, 0.3%-5.9%) in the late group. The full-term reference group showed a higher prevalence of overweight (9.0%; 95% CI, 5.1%-14.6%) and obesity (3.5%; 95% CI, 1.3%-7.5%).

**Conclusions and Relevance:**

In this randomized clinical trial of preterm infants, initiating complementary feeding between corrected age 12 and 17 weeks did not affect overweight and obesity prevalence at corrected age 2 years.

**Trial Registration:**

Onderzoekmetmensen.nl Identifier: NL-OMON53076

## Key Points

**Question:**

What is the effect of initiating complementary feeding at corrected age 12 weeks vs 17 weeks on the prevalence of overweight and obesity at corrected age 2 years in preterm infants?

**Findings:**

In this randomized clinical trial of 255 preterm infants, there was no significant difference in the prevalence of overweight and obesity at corrected age 2 years between preterm infants initiating complementary feeding at corrected age 12 weeks vs 17 weeks.

**Meaning:**

These findings suggest that the timing of complementary feeding in preterm infants does not affect the risk of overweight, including obesity.

## Teaser

This randomized clinical trial investigates the effect of initiating complementary feeding in preterm infants at corrected age 12 weeks vs 17 weeks on prevalence of overweight and obesity at corrected age 2 years.

## Introduction

The rising prevalence of childhood overweight and its associated health implications have become a major concern globally.^1^ While the factors contributing to this trend are multifaceted, the understanding of possible causes is crucial. Among these factors, early-life nutrition has emerged as a determinant of growth trajectories and long-term health outcomes, including the risk of overweight and obesity.

Complementary feeding marks a crucial transition in an infant’s early-life nutrition and should be both timely and adequate to meet the evolving nutritional needs of young infants.^2^ The World Health Organization (WHO) defines complementary feeding as the introduction of solid foods alongside breast milk or formula and advises that full-term infants start complementary feeding after age 6 months.^3^ The European Society for Paediatric Gastroenterology, Hepatology and Nutrition Committee on Nutrition has advised that for healthy full-term infants, starting complementary feeding between age 4 and 6 months is safe and does not negatively influence growth during infancy or early childhood. The committee’s advice is promoted by the child health clinics in the Netherlands.^4,5^

For preterm infants, guidelines on the optimal timing for initiating complementary feeding are lacking.^6,7,8^ Preterm infants (born before gestational age [GA] 37 weeks) represent a vulnerable population with additional nutritional needs. Approximately 10% of infants worldwide are born preterm, constituting a substantial portion of the global population.^9^

Observational studies have shown that preterm infants often initiate solid foods earlier,^8,10,11,12,13^ but it remains unclear whether this is associated with an increased risk of overweight,^6,13,14^ as observed in full-term infants.^15,16,17^ For very low-birth-weight preterm infants, a recently published randomized clinical trial showed that introducing a standardized complementary diet at 2 different time points did not have a persistent effect on anthropometric parameters at age 1 year.^18^ Nevertheless, a transient short-term effect on weight *z* scores at 6 months was observed, indicating a more rapid weight gain in the early complementary diet group, which may serve as a risk factor for the development of overweight. The aim of our randomized clinical trial was to investigate the effect of initiating complementary feeding in moderate- to late-preterm infants at corrected age 12 weeks vs 17 weeks on the prevalence of overweight and obesity at corrected age 2 years, considering potential implications for metabolic health and long-term outcomes.

## Methods

### Study Design

This randomized clinical trial with a parallel design was performed at 17 hospitals in the Netherlands. Preterm infants were randomized to receive complementary feeding at corrected age 12 weeks or 17 weeks (give or take 1 week). A full-term infant group served as a reference. The study was conducted between May 13, 2016, and April 26, 2021, with follow-up completed in December 2023. This trial was approved by the ethics committee of Amsterdam UMC. Written informed consent was obtained from both parents or from caregivers prior to the infants’ inclusion in the study. The results are reported in accordance with the Consolidated Standards of Reporting Trials (CONSORT) statement. The full protocol is provided in Supplement 1.

### Participants

Infants born between GA 30 and 36 weeks were eligible. Exclusion criteria were small for GA (birthweight <2.3 percentile); intestinal disorders interfering with a stable growth; moderate to severe bronchopulmonary dysplasia; congenital heart disease (hemodynamic consequences); severe cow milk allergy; congenital anomalies of the ear, nose, throat, esophagus, or tracheal area needing surgical correction; syndrome disorders; intraventricular hemorrhage grade 3 or 4 (following the criteria of Volpe et al^19^); other diseases deemed to interfere with stable growth; initiation of complementary feeding before randomization; and not understanding the Dutch language.

The full-term infants born after GA 37 weeks had the same exclusion criteria. They were included from 10 different hospitals in the Netherlands.

### Randomization

The preterm infants were randomized at corrected age 10 weeks using computer-generated randomization (Castor Electronic Data Capture), with a 1:1 allocation ratio and stratification by hospital. Block randomization with blocks of 2, 6, and 8 was applied. Twins and triplets were assigned to the same group. The parents of the infants, medical staff, and researchers were not masked to the randomization. The full-term infants were not randomized and could start complementary feeding anytime.

### Intervention

Complementary feeding was defined by the WHO as introducing non-(breast)milk foods or nutritive liquids.^20^ The feeding pattern followed the advice of the child health clinics in the Netherlands. The exact date of starting complementary feeding was recorded via a standardized questionnaire.

### Outcome

The primary outcome was the prevalence of overweight and obesity at corrected age 2 years as determined by body mass index (BMI) cutoff values set by the International Obesity Task Force (IOTF)^21^ and according to the WHO definition.^22^ Other anthropometric outcomes were height, weight, head circumference, BMI, and corresponding *z* scores at corrected age 1 and 2 years. Secondary outcomes were Scoring of Atopic Dermatitis (SCORAD) index,^23^ neurodevelopment, and health-related quality of life between the 2 groups of preterm infants. The SCORAD index contains multiple components, including (A) extension of lesions (maximum of 100 points), (B) clinical signs (maximum of 18 points), and (C) symptoms (maximum of 20 points) and is calculated as SCORAD = (A / 5) + (7B / 2) + C. Health-related quality-of-life scores were transformed on a scale of 0 to 100 points. Before term age, Fenton growth charts were used for anthropometrics.^24^ At corrected age 1 and 2 years, *z* scores were calculated using the WHO’s Multicentre Growth Reference Study growth standards.^22,25^

### Procedures

For preterm infants, anthropometric measurements and SCORAD^23^ were conducted at randomization and corrected age 1 and 2 years. Neurodevelopment was measured using the Ages and Stages Questionnaire^26,27^ at corrected age 1 and 2 years. Health-related quality of life was assessed using the Pediatric Quality of Life Infant Scales (13-24 months)^28^ at corrected age 2 years. For the full-term infants, the same anthropometric measurements were performed at age 3 months, 1 year, and 2 years.

For all infants, the parents maintained a dietary record before the start of complementary feeding and directly after the start of complementary feeding, including details on milk feeding and the introduction of complementary food. Eating behavior was measured using the Baby Eating Behavior Questionnaire^22^ before the start of complementary feeding.

### Sample Size

The sample size calculation for this study was based on previously reported effects. Jingxiong et al^29^ reported that an early start of complementary feeding was associated with an odds ratio (OR) of 1.76 for developing overweight. A study by Young et al^30^ reported that excess weight gain between 4 and 6 months was associated with an OR of 2.5 for overweight at 18 to 24 months.^31^ On the basis of these studies, we used an OR of 2.3. With a slightly lower prevalence of 6% (compared with 8% based on the IOTF cutoff values^31^) in preterm infants, a sample size of 275 patients per group was needed for 80% power at a .05 significance level, as calculated using G*Power, version 3.^32,33^ With a 5% loss to follow-up, 600 children were targeted (300 per group). We deemed a reference group of approximately 150 full-term children to be sufficient.

### Statistical Analysis

The data were analyzed on an intention-to-treat basis. The baseline characteristics were reported per group, including GA, birth weight, sex, cesarean delivery, and feeding for infants and self-reported parental age, sex, BMI, highest education attained, and ethnicity (Dutch and other). Ethnicity was included to provide insight into the study population and the degree of generalizability. In total, there were 20 different countries of origin. Dependencies between twins were not taken into account in the descriptive analysis. The primary outcome was analyzed as a dichotomous variable, combining infants with overweight and obesity. Logistic mixed models with hospital site and twins as random intercepts were used. The other anthropometric outcomes were assessed analogous to the primary analysis using linear mixed models, taking into account the repeated measurements per participant and hospital site and twins as random intercepts. Student *t* test was used to analyze the secondary normally distributed continuous variables, and Mann-Whitney *U* test was used in cases of skewed distributions. Differences among categorical variables were tested with χ^2^ tests unless any expected value was less than 5, in which case Fisher exact test was used. A per-protocol analysis was performed for preterm infants who strictly adhered to the study protocol. The statistical analysis was performed using SPSS, version 29.0 (IBM Corp) and Stata/SE, version 18.0 (StataCorp LLC). The threshold for significance was set at a 2-sided *P* < .05.

## Results

**Figure 1.**
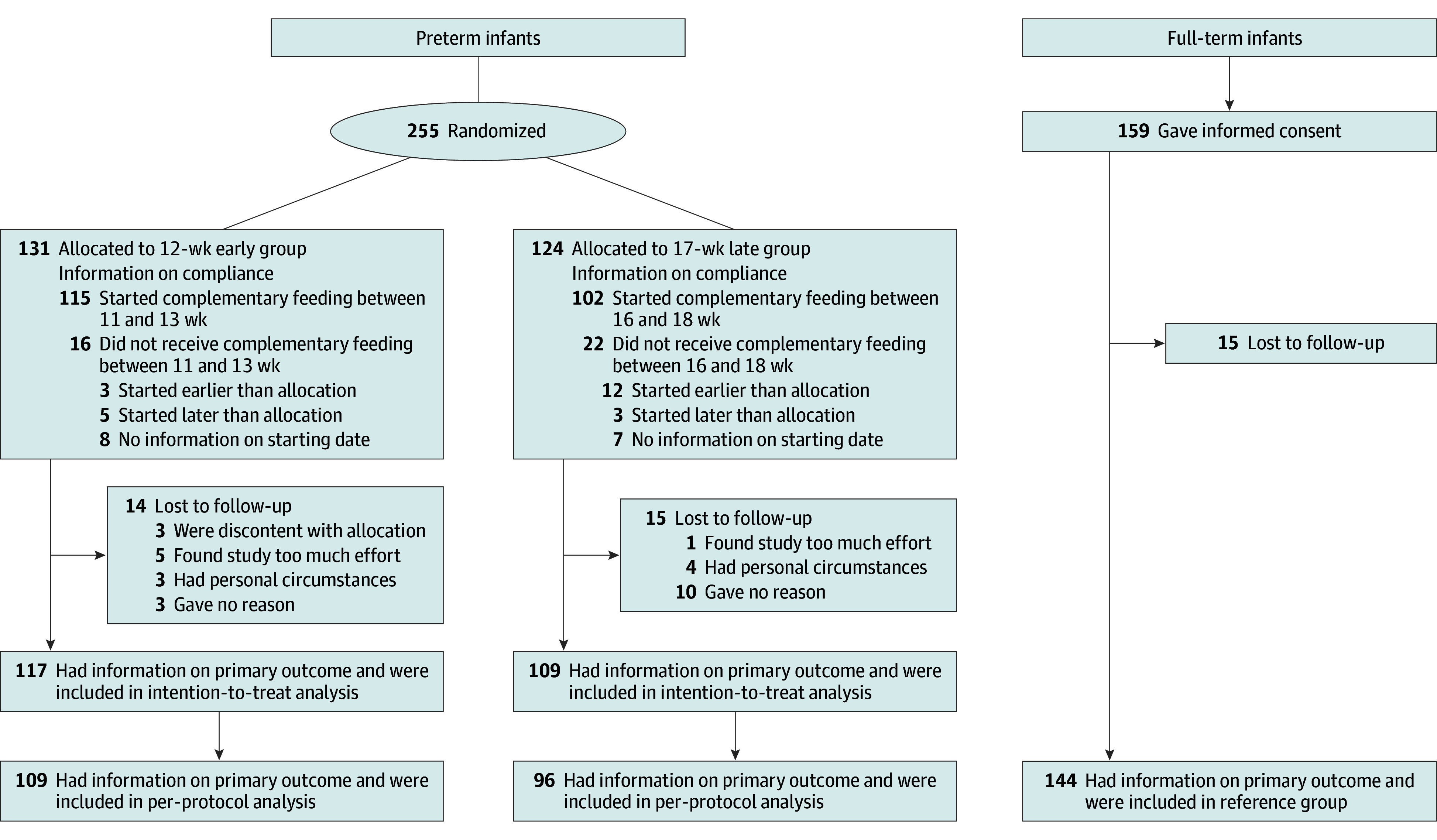
Consolidated Standards of Reporting Trials Flow Diagram of Preterm and Full-Term Infants of the SPOON Study

A total of 255 preterm infants were included and randomly assigned, with 131 (51.4%; median [IQR] GA, 34 weeks 2 days [32 weeks 5 days to 35 weeks 1 day]; 54 female [41.2%] and 77 male [58.8%]) allocated to the early group and 124 (48.6%; median [IQR] GA, 34 weeks 0 days [32 weeks 6 days to 34 weeks 6 days]; 62 female [50.0%] and 62 male [50.0%]) allocated to the late group (Figure 1), representing approximately 10% of eligible preterm infants. Despite many attempts to improve the recruitment rate, we failed to include 600 preterm infants within a period of 5 years. We had to stop the trial as the funding budget did not allow for a continuation of prolonged recruitment with a subsequent additional follow-up period of 2 years.

A total of 159 full-term infants (median [IQR] GA, 40 weeks 0 days [39 weeks 0 days to 41 weeks 0 days]; 84 female [52.8%] and 75 male [47.2%]) were included, so the total number of included children was 414. The assessment of the primary outcome at corrected age 2 years was possible for 226 preterm infants (88.6%). For the full-term infants, 144 (90.6%) had recorded information on both weight and height at age 2 years. The baseline characteristics of the participating preterm infants at birth and before randomization were similar for the early and late groups (Table 1; eTable 1 in Supplement 2). One pair of twins in the late group was excluded because of dysmaturity. The full-term infants naturally differed from preterm infants in GA and birth weight. Additionally, preterm infants were more often born by cesarean delivery and less likely to have 1 or both parents of Dutch ethnicity compared with full-term infants, and no infants in the full-term reference group were twins.

**Table 1.** Baseline Characteristics of the SPOON Study Population

Characteristic	Patients, No. (%)		
	Preterm infants		Term infants
	Early group	Late group	
**Patients**			
No. of patients	131 (51.4)	124 (48.6)	159 (100)
Gestational age, median (IQR)	34 wk 2 d (32 wk 5 d to 35 wk 1 d)	34 wk 0 d (32 wk 6 d to 34 wk 6 d)	40 wk 0 d (39 wk 0 d to 41 wk 0 d)
Birth weight, mean (SD), g	2128 (488)	2096 (473)	3735 (478)
Twins	26 (19.8)	23 (18.5)	0
Sex			
Female	54 (41.2)	62 (50.0)	84 (52.8)
Male	77 (58.8)	62 (50.0)	75 (47.2)
Cesarean delivery	42 (32.1)	51 (41.1)	27 (17.0)
Feeding^a^			
Exclusively breast	28 (22.8)	23 (19.5)	73 (45.9)
Breast and formula combination	16 (13.0)	21 (17.8)	21 (13.2)
Exclusively formula	79 (64.2)	74 (62.7)	62 (39.0)
**Parents**			
Age, mean (SD), y^b^			
Mother	31.7 (4.6)	31.2 (4.5)	31.3 (4.4)
Father	34.4 (5.9)	33.1 (4.8)	33.9 (5.2)
BMI, median (IQR)^b^			
Mother	24.4 (22.1 to 27.6)	24.09 (21.7 to 26.7)	25.3 (22.7 to 28.4)
Father	24.9 (22.9 to 26.3)	25.18 (22.9 to 27.5)	24.7 (22.9 to 26.7)
Highest education^b^			
Mother			
Secondary school	6 (4.6)	11 (8.9)	16 (10.1)
Vocational education and training	40 (30.5)	38 (30.6)	48 (30.2)
Higher education	83 (63.4)	74 (59.7)	93 (58.5)
Father			
Secondary school	24 (18.3)	22 (17.7)	26 (16.4)
Vocational education and training	36 (27.5)	40 (32.3)	47 (29.6)
Higher education	65 (49.6)	56 (45.2)	84 (52.8)
Ethnicity^b^			
Mother			
Dutch	104 (79.4)	104 (83.9)	147 (92.5)
Other^c^	12 (9.2)	8 (6.5)	10 (6.3)
Father			
Dutch	105 (80.2)	98 (79.0)	150 (94.3)
Other^c^	8 (6.1)	9 (7.3)	7 (4.4)

Abbreviation: BMI, body mass index (calculated as weight in kilograms divided by height in meters squared).

^a^ During the first 3 months.

^b^ Information was self-reported by the parents in questionnaires at randomization.

^c^ Countries of origin included Aruba, Bulgaria, Curacao, Dominican Republic, Eritrea, Estonia, France, Germany, Ghana, Italy, Morocco, Poland, South Africa, Spain, Suriname, Turkey, United Kingdom, US, Yugoslavia, and Zimbabwe.

### Primary Outcome

At corrected age 2 years, the prevalence of overweight, based on the IOTF cutoff values, was 6.0% (95% CI, 2.7%-11.5%) in the early group and 5.5% (95% CI, 2.3%-11.1%) in the late group, and the prevalence of obesity was 1.7% (95% CI, 0.3%-5.5%) in the early group and 1.8% (95% CI, 0.3%-5.9%) in the late group, indicating no significant difference between the groups (*P* = .97). According to the WHO definition, no significant differences were found in prevalence of overweight and obesity between groups (2.6% and 0.9%, respectively, in the early group and 5.5% and 0%, respectively, in the late group; *P* = .34).

Based on a logistic mixed model, the OR for overweight and obesity associated with early complementary feeding was 1.05 (95% CI, 0.39-2.83; *P* = .92) (Table 2, model 1). With adjustment for the dependency of twins and hospital, the results remained unchanged (Table 2, model 2). Furthermore, no significant differences were observed between the early and late groups in any other anthropometric measurements at corrected age 1 and 2 years (Table 2; eTable 2 in Supplement 2).

**Table 2.** Growth Parameters of Preterm Infants at Corrected Age 2 Years, Including Mixed Model for Repeated Measurements

Growth parameter	Preterm infants, mean (SD)		Between-group mean difference (95% CI)	*P* value	Model				Term infants, mean (SD)
	Early group	Late group			1^a^		2^b^		
					Regression coefficient (95% CI)	*P* value	Regression coefficient (95% CI)	*P* value	
Height, cm	87.34 (3.63)^c^	87.59 (3.76)^d^	−0.25 (−1.2 to 0.72)	.62	0.49 (−2.41 to 3.39)	.74	1.21 (−1.48 to 3.90)	.38	88.54 (3.28)^e^
Height-for-age *z* score	0.15 (1.19)^c^	0.16 (1.13)^d^	−0.01 (−0.32 to 0.29)	.93	−0.10 (−0.30 to 0.10)	.34	−0.09 (−0.30 to 0.11)	.37	0.42 (0.95)^e^
Weight, kg	12.44 (1.50)^f^	12.52 (1.58)^d^	−0.08 (−0.48 to 0.32)	.70	−0.007 (−0.46 to 0.45)	.98	−0.007 (−0.46 to 0.45)	.98	13.07 (1.46)^g^
Weight-for-age *z* score	0.32 (1.09)^f^	0.36 (1.02)^d^	−0.03 (−0.31 to 0.24)	.80	−0.09 (−0.27 to 0.10)	.36	−0.10 (−0.30 to 0.11)	.37	0.68 (0.86)^g^
Weight-for-height *z* score	0.29 (1.11)^c^	0.34 (0.94)^d^	−0.05 (−0.32 to 0.22)	.73	−0.09 (−0.27 to 0.10)	.36	−0.07 (−0.29 to 0.15)	.53	0.61 (0.96)^h^
Head circumference, cm	49.14 (1.46)^i^	48.63 (1.94)^j^	0.51 (−0.16 to 1.19)	.14	7.20 (−6.08 to 20.47)	.29	6.21 (−6.98 to 19.39)	.36	49.82 (5.02)^k^
Head circumference-for-age *z* score	0.91 (1.05)^i^	0.67 (1.29)^j^	0.25 (−0.22 to 0.71)	.30	−0.04 (−0.31 to 0.23)	.78	0.06 (−0.17 to 0.29)	.61	1.03 (0.99)^k^
BMI	16.28 (1.50)^c^	16.28 (1.32)^d^	0.002 (−0.37 to 0.37)	.99	−0.04 (−0.29 to 0.22)	.77	−0.05 (−0.34 to 0.23)	.72	16.68 (1.44)^k^
BMI-for-age *z* score	−0.22 (1.25)^c^	−0.15 (1.03)^d^	−0.07 (−0.37 to 0.23)	.65	−0.08 (−0.26 to 0.11)	.42	−0.08 (−0.29 to 0.12)	.43	0.61 (0.99)^k^
**Weight according to IOTF definition**									
Normal, No. (%)	108 (92.3)^c^	101 (92.7)^d^	NA	.92	1.05 (0.39 to 2.83)^l^	.92	1.03 (0.36 to 2.81)^l^	.96	126 (87.5)^h^
Overweight and obesity, No. (%)	9 (7.7)^c^	8 (7.3)^d^	NA						18 (12.5)^h^
**Weight according to WHO definition**									
Normal, No. (%)	113 (96.6)^c^	103 (94.5)^d^	NA	.45	0.61 (0.17 to 2.21)^l^	.45	0.59 (0.16 to 2.22)^l^	.44	132 (91.7)^h^
Overweight and obesity, No. (%)	4 (3.4)^c^	6 (5.5)^d^	NA						12 (8.3)^h^

Abbreviations: BMI, body mass index (calculated as weight in kilograms divided by height in meters squared); IOTF, International Obesity Task Force; NA, not applicable; WHO, World Health Organization.

^a^ Model 1, mixed model for repeated measurements without intercept.

^b^ Model 2, mixed model for repeated measurements with correction for individual, hospital site, and twins.

^c^ Infants with complete data, n = 117.

^d^ Infants with complete data, n = 109.

^e^ Infants with complete data, n = 146.

^f^ Infants with complete data, n = 119.

^g^ Infants with complete data, n = 147.

^h^ Infants with complete data n = 144.

^i^ Infants with complete data, n = 56.

^j^ Infants with complete data, n = 44.

^k^ Infants with complete data, n = 67.

^l^ Odds ratio (95% CI).

**Figure 2.**
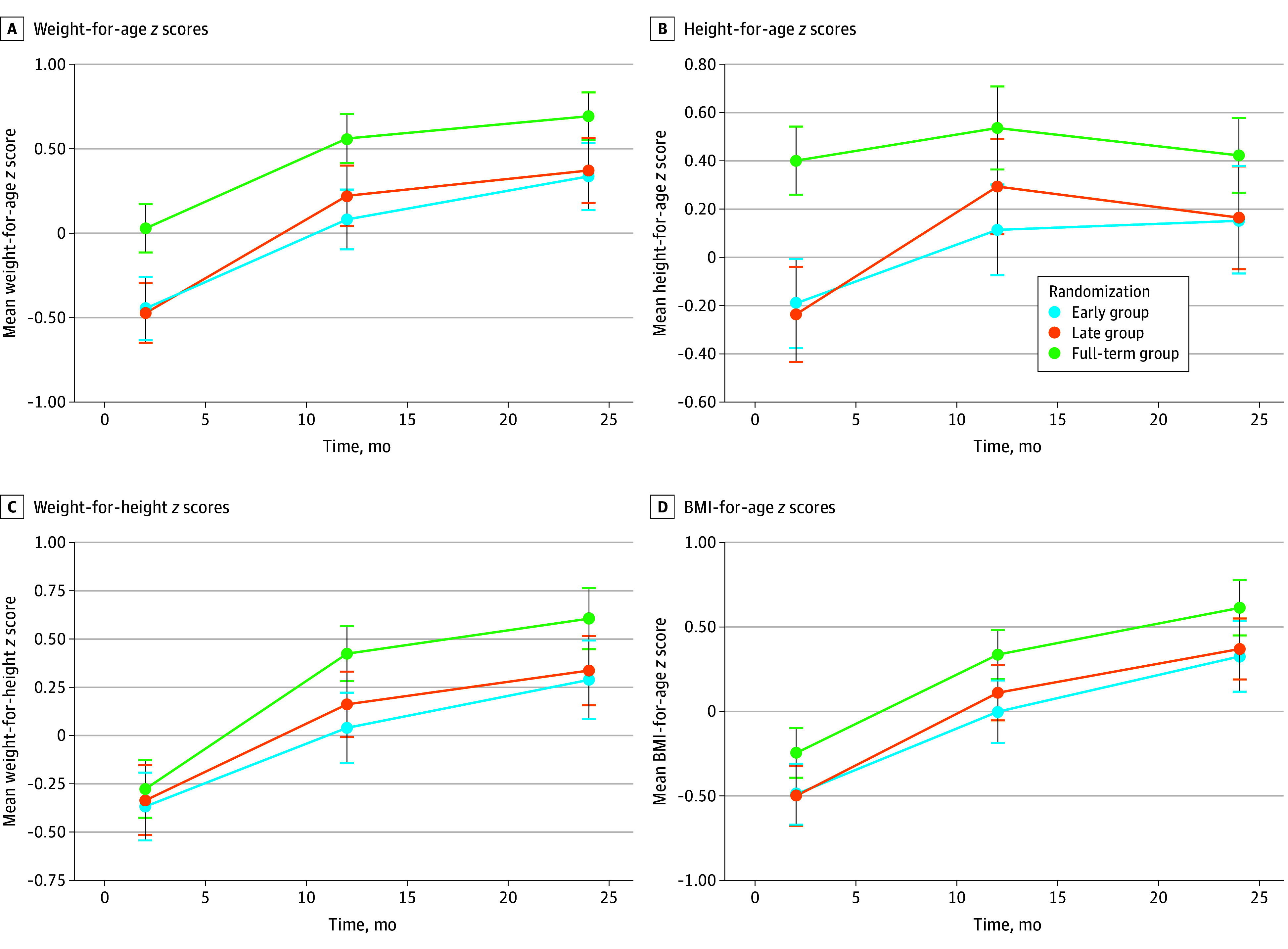
Change in Growth Parameters Over Time Until (Corrected) Age 2 Years Time is corrected for age of preterm infants. Error bars indicate 95% CIs.

The full-term reference group showed a higher prevalence for overweight (9.0%; 95% CI, 5.1%-14.6%) and obesity (3.5%; 95% CI, 1.3%-7.5%). The full-term infants followed a similar growth pattern, with a higher mean weight, height, head circumference, BMI, and corresponding *z* scores throughout the entire study period (Figure 2; eFigure in Supplement 2).

### Secondary Outcomes

Health-related quality-of-life scores showed a significant difference in the physical symptoms category at corrected age 2 years (early group, 87.26 points [IQR, 82.50-95.00 points]; late group, 87.50 points [IQR, 80.00-95.00 points]; *P* = .05) (eTable 3 in Supplement 2). Specifically, the self-reported physical symptoms of diarrhea and constipation were reported significantly more frequently as almost always in the early group compared with the late group (diarrhea, 50.9% vs 29.1%; constipation, 77.2% vs 61.2%; *P* = .02) at corrected age 2 years.

Neurodevelopment scores were similar at corrected age 1 and 2 years between the 2 preterm groups (eTable 4 in Supplement 2). The SCORAD index revealed a higher proportion with a moderate score (25-50) at corrected age 1 year in the early group (6 infants [6.3%] vs 0 infants in the late group; *P* = .01) (eTable 5 in Supplement 2). However, this difference was no longer observed at corrected age 2 years.

The majority of the preterm infants started complementary feeding with vegetables as their first food. Vegetables as the first food was significantly more common in the early group compared with the late group (114 infants [92.7%] vs 91 infants [77.8%]; *P* = .001) (eTable 6 in Supplement 2). Within the full-term infant group, 106 (68.8%) started with vegetables. Additional correction for the type of food at the initiation of complementary feeding did not result in any significant difference among the groups across all anthropometric parameters.

### Dropouts and Per-Protocol Analysis

When comparing the baseline characteristics of the dropouts (n = 29) with the analytic sample (n = 226), we found a significantly higher percentage of twins in the dropout group (12 [41.4%] vs 37 [16.4%]; *P* = .001). For the per-protocol analysis, the primary outcome was available for 109 infants from the early group and 96 from the late group (Figure 1). This analysis yielded results consistent with the intention-to-treat analysis, showing comparable prevalence of overweight and obesity (IOTF cutoff values) in the early group (6.4% and 0.9%, respectively) vs the late group (4.2% and 2.1%, respectively) (*P* = .62), as well as showed no significant differences in any of the anthropometric measurements and corresponding *z* scores at corrected age 2 years (eTable 7 in Supplement 2).

## Discussion

This randomized clinical trial shows no difference in the prevalence of overweight and obesity at corrected age 2 years between preterm infants starting complementary feeding at corrected age 12 weeks or 17 weeks. All preterm infants had a lower weight-for-height *z* score and BMI *z* score than the full-term infant reference group throughout the study.

To date, 3 randomized clinical trials have investigated the time of initiating complementary feeding in preterm infants and the effect on growth.^18,34,35^ Marriott et al^34^ compared the effect of early initiation of complementary feeding, with higher energy and protein content, after postnatal age 13 weeks with late initiation of complementary feeding (current best practice) after postnatal age 17 weeks in 68 preterm infants. They reported a significantly higher mean rate of growth in length per week in the early group compared with the late group after corrected age 12 months (5.1 vs 4.9 mm/wk; *P* = .04). Today, postdischarge formula and fortification are standard practices, which make the results of Marriott et al outdated.

The study of Gupta et al^35^ investigated the effect of initiation of complementary feeding at 4 months vs 6 months on growth at corrected age 12 months in 373 preterm infants born before GA 34 weeks. At corrected age 12 months, no significant effect on weight-for-age *z* score or the other growth parameters was found. This study was performed in India; thus, the authors’ rationale for starting complementary feeding late may have been for hygienic reasons. Despite the difference in the classification of early and late introduction of complementary feeding, both Gupta et al and our study emphasize that complementary feeding can be initiated from 12 weeks after term onward.

Haiden et al^18^ conducted a study involving preterm infants with a birth weight of less than 1500 g who received a standardized complementary diet during the whole first year of life starting at either corrected age 10 to 12 weeks or 16 to 18 weeks. They initiated complementary feeding at the same time points as our study but focused on height at 12 months, corrected for term, as a reliable estimator for skeletal growth and fat-free body mass. They found no significant difference between the early and late complementary feeding groups for various anthropometric measurements.^18^ Hence, we expanded their findings in growth parameters in the first 2 years to late preterm infants.

Altogether, combining the data from previous trials and ours, the results suggest that the timing of the start of complementary feeding does not affect body weight indices when initiated between corrected age 12 and 17 weeks. This finding is reassuring for parents and children as they can start within this time frame as mutually desired.

### Strengths and Limitations

A strength of our study is that we specifically focused on moderate- to late-preterm infants, the largest group of preterm infants born worldwide.^9^ Much of the research has been devoted to extremely preterm infants and limited by low numbers of children involved. Furthermore, our study derived results from daily clinical practice. Child health clinics affiliated with 17 hospitals across the Netherlands advised parents on when to start complementary feeding, indicating generalizability to the moderate- to late-preterm populations of high-income countries. Another strength is that the timing of complementary feeding did not influence the occurrence of atopic dermatitis or health-related quality of life throughout the infants’ first 2 years. In addition to growth, this study examined neurodevelopment at corrected age 1 and 2 years. We found no significant difference between the 2 groups, indicating that the preterm infants in both groups were comparable in a broader context. The neurodevelopment of our preterm infants at corrected age 2 years showed similar levels of Ages and Stages Questionnaire neurodevelopment as a group of 703 preterm infants from Flamant et al,^36^ indicating the generalizability of our preterm study population.

Our study also had several limitations. The trial did not meet the preconceived numbers of inclusion. Only 10% of the parents of the eligible preterm infants expressed interest in participating in the current study mostly because they preferred to decide for themselves when to start complementary feeding rather than having this determined by randomization. Furthermore, given delays caused by the COVID-19 pandemic, recruitment would have had to last at least until 2028, with a study closure date of 2030, to achieve the estimated sample size, which was financially not feasible. Consequently, the underpowered nature of the trial raises important questions about the interpretation of the findings. However, considering the minimal difference in effect between the 2 groups, the likelihood of detecting a statistically significant difference with clinical relevance, even if the study was adequately powered, is very low. Therefore, the results of this randomized clinical trial do not necessarily diminish its potential clinical relevance. Furthermore, despite being terminated early, this study is still, to our knowledge, the largest multicenter investigation performed in a high-income country and the second largest randomized clinical trial performed on this topic.

In addition to addressing the risk of overweight and obesity, it is imperative to consider a multitude of other factors in the discussion on the optimal timing of complementary feeding in preterm infants. Among these factors are neurodevelopmental skills and the presence of possible comorbidities in preterm infants.^14^ Furthermore, the behavior of the infant, emotional factors of the parents, and the risk for allergies or atopic manifestations should also be assessed. Additionally, attention to the intake of essential micronutrients is paramount, as preterm infants often have specific nutritional requirements that must be met to support optimal growth and development. Therefore, while addressing overweight is important, it is essential to adopt a holistic approach to nutritional management in preterm infants. By addressing these multifaceted aspects, we may optimize the nutritional care of preterm infants and promote their long-term health outcomes.

## Conclusions

In this randomized clinical trial of preterm infants, initiating complementary feeding between corrected age 12 and 17 weeks did not increase the risk of overweight or obesity. The findings showed no discernible impact on growth outcomes, including overweight and obesity, providing parents of moderate- to late-preterm infants, along with their pediatricians or general practitioners, the flexibility to choose a feeding timeline within this range that aligns with their preferences, circumstances, and the developmental needs of the infant.

## Notes

Supplement 1.
Trial Protocol

Supplement 2. **eTable 1.** Characteristics of the Study Population Before Randomization (for Preterm Infants) or at 3 Months (for Full-Term Infants) **eTable 2.** Growth Parameters of Preterm Infants at Corrected Age 1 Year, Including Mixed Model With Correction for Correlations **eTable 3.** Health-Related Quality of Life: PedsQL Infant Scales **eTable 4.** Neurodevelopment: ASQ-3 at Correct Age 1 Year and 2 Years for Preterm Infants **eTable 5.** SCORAD for Atopic Dermatitis in Preterm Infants **eTable 6.** Initiating Complementary Feeding **eTable 7.** Per-Protocol Analysis: Growth Parameter of Preterm Infants at Correct Age 2 Years, Including Mixed model for Repeated Measurements **eFigure.** Change in Growth Parameters Over Time Until (Corrected) Age 2 Years for Preterm and Full-Term Infants
eReference.

Supplement 3.
Nonauthor Collaborators

Supplement 4.
Data Sharing Statement
