# Supplementary material for: Timing of Complementary Feeding in Preterm Infants and Prevalence of Overweight and Obesity: A Randomized Clinical Trial
Source: JAMA Netw Open. 2025 Apr 30;8(4):e252968. doi: 10.1001/jamanetworkopen.2025.2968 (PMC12044495; doi:10.1001/jamanetworkopen.2025.2968)
Supplement: Supplement 1. — Trial Protocol [file jamanetwopen-e252968-s001.pdf]

SPOON

SOLID FOOD IN PRETERM INFANTS AND THE EFFECT ON OBESITY IN THE NETHERLANDS

Timing of complementary feeding in preterm infants

RESEARCH PROTOCOL

PROTOCOL TITLE: Solid foods in Preterm infants and the effect On Obesity in the Netherlands (SPOON-study)

**Protocol ID**	**ZGV-kindergeneeskunde-01**
**Short title**	**Timing of complementary feeding in preterm infants**
**Version**	**14**
**Date**	**04-05-2022**
**Coordinating investigator/project leader**	**dhr. Prof. Dr. J.B. van Goudoever**<br>**VUmc**<br>**De Boelelaan 1117**<br>**1081 HV Amsterdam**<br>**E-mail:** h.vangoudoever@amsterdamumc.nl

**Principal investigator(s)**<br>**(in Dutch:**<br>**hoofdonderzoeker/uitvoerder)**	**1. dhr. Prof. Dr. J.B. van Goudoever**<br>**VUmc**<br>**De Boelelaan 1117**<br>**1081 HV Amsterdam**<br>**E-mail:** h.vangoudoever@amsterdamumc.nl<br>**2. Mw. dr. A. J. Janse**<br>**Ziekenhuis Gelderse Vallei,**<br>**secretariaat kindergeneeskunde.**<br>**Willy Brandtlaan 10, 6716 RP Ede**<br>**Telefoon: (0318) 435092**<br>**E-mail:** JanseA1@zgv.nl<br>**3. mw. Prof. Ir. E.J.M. Feskens**<br>**Division of Human Nutrition, Wageningen University**<br>**PO box 8129, 6700 EV Wageningen**<br>**E-mail:** Edith.Feskens@wur.nl<br>**4. Mw. drs. K.M. Vissers**<br>**Ziekenhuis GelderseVallei,**<br>**Secretariaat kindergeneeskunde**<br>**Willy Brandtlaan 10, 6716 RP Ede**<br>**Telefoon : (0318) 435092**<br>**E-mail:** VissersK@zgv.nl
**Sponsor (in Dutch: verrichter/opdrachtgever)**	**VUmc**<br>**De Boelelaan 1117**<br>**1081 HV Amsterdam**
**Subsiding party**	**Nutricia Nederland**<br>**M. Miskiewicz financieel directeur Early Life Nutrition Nutricia Nederland B.V.**
**Independent physician(s)**	**Dr. M. Weinans**<br>**Ziekenhuis Gelderse Vallei, secretariaat gynaecologie.**<br>**Willy Brandtlaan 10, 6716 RP Ede**<br>**Telefoon: (0318) 435175**

	E-mail: WeimansM@zgv.nl
**Laboratory sites**	
**Pharmacy <*if applicable*>**	**Not applicable**

PROTOCOL SIGNATURE SHEET

Name	Signature	Date
**Sponsor or legal representative:**<br>***mw. Drs. M. Koppejan-Stapel***<br>***Head of Department Pediatrics***<br><br>**For non-commercial research**	**Not applicable (non-commercial research)**	
**Coordinating Investigator/Project leader/**<br>**dhr. Prof. Dr. J.B. van Goudoever**		
**Principal Investigator:**<br>**dhr. Prof. Dr. J.B. van Goudoever**		

TABLE OF CONTENTS

SUMMARY 10
1. INTRODUCTION AND RATIONALE 11
1.1 Complementary feeding 11
1.1.1 Complementary feeding in preterm infants: the benefits and risks 11
1.1.2 The start of complementary feeding and developing obesity 13
1.1.3 Current advice for the timing of complementary feeding in preterm infants 15
2. OBJECTIVES 17
3. STUDY DESIGN 19
4. STUDY POPULATION 22
4.1 Population (base) 22
4.1.1 Inclusion criteria 22
4.1.2 Exclusion criteria 22
4.2 Control population 23
4.3 Sample size calculation 24
5. TREATMENT OF SUBJECTS 25
5.5 Investigational product/treatment 25
5.6 Use of co-intervention (if applicable) 25
5.7 Escape medication (if applicable) 26
6. INVESTIGATIONAL PRODUCT 26
6.1 Name and description of investigational product(s) 26
6.2 Summary of findings from non-clinical studies 26
6.3 Summary of findings from clinical studies 26
6.4 Summary of known and potential risks and benefits 26
6.5 Description and justification of route of administration and dosage 26
6.6 Dosages, dosage modifications and method of administration 26
6.7 Preparation and labelling of Investigational Medicinal Product 26
6.8 Drug accountability 26
7. NON-INVESTIGATIONAL PRODUCT 26
7.1 Name and description of non-investigational product(s) 27
7.2 Summary of findings from non-clinical studies 27
7.3 Summary of findings from clinical studies 27
7.4 Summary of known and potential risks and benefits 27
7.5 Description and justification of route of administration and dosage 27
7.6 Dosages, dosage modifications and method of administration 27
7.7 Preparation and labelling of Non Investigational Medicinal Product 27
7.8 Drug accountability 27
8. METHODS 28
8.1 Study parameters/endpoints 28
8.1.1 Main study parameter/endpoint 28
8.1.2 Secondary study parameters/endpoints 28
8.1.3 Other study parameters (if applicable) 28

8.1.4 Study parameters tested in controls 28
8.2 Randomisation, blinding and treatment allocation 29
8.3 Study procedures 29
8.3.1 Height – Standard care 29
8.3.2 Weight – Standard care 29
8.3.3 Body Mass Index (BMI) – Standard care 29
8.3.4 SCORAD Index – Extra in this study 30
8.3.5 Health Related Quality of Life (HRQoL): Infant Toddler Quality of Life Questionnaire (ITQoL) or Pediatric Quality of Life Inventory (PedsQL) – Extra in this study 30
8.3.6 Eating behavior – Extra in this study 31
8.3.7 Child Feeding Questionnaire (CFQ) – Extra in this study 31
8.3.8 Ages and Stages Questionnaire (ASQ) – Extra in this study 32
8.3.9 Dietary intake – Extra in this study 32
8.4 Withdrawal of individual subjects 33
8.5 Replacement of individual subjects after withdrawal 33
8.6 Follow-up of subjects withdrawn from treatment 33
8.7 Premature termination of the study 33
9. SAFETY REPORTING 35
9.1 Section 10 WMO event 35
9.2 Adverse and serious adverse events 35
9.3 Follow-up of adverse events 35
9.4 Data Safety Monitoring Board (DSMB) 36
10. STATISTICAL ANALYSIS 37
11. ETHICAL CONSIDERATIONS 38
11.1 Regulation statement 38
11.2 Recruitment and consent 38
11.3 Objection by minors or incapacitated subjects (if applicable) 38
11.4 Benefits and risks assessment, group relatedness 38
11.5 Compensation for injury 39
11.6 Incentives (if applicable) 40
12. ADMINISTRATIVE ASPECTS AND PUBLICATION 41
12.1 Handling and storage of data and documents 41
12.2 Monitoring and Quality Assurance 41
12.3 Amendments 41
12.4 Annual progress report 41
12.5 End of study report 41
12.6 Public disclosure and publication policy 42

LIST OF ABBREVIATIONS AND RELEVANT DEFINITIONS

ADP	Air Displacement Plethysmography
APGAR	Score to quickly and summarily assess the health of the newborn
ANOVA	Analysis of variance
BEBQ	Baby Eating Behaviour Questionnaire
BIA	Body impedance
BMI	Body Mass Index
BOD POD	An air displacement plethysmography system to determine body composition
BPD	Bronchopulmonary dysplasia
CCMO	Centrale Commissie Mensgebonden Onderzoek
CFQ	Child Food Questionnaire
CRP	C-reactive protein
Cm	Centimeter
DSMB	Data Safety Monitoring Board
ENT	Ear Nose Throat
ESPGHAN	European Society for Paediatric Gastroenterology, Hepatology and Nutrition
HC	Head Circumference
HRQoL	Health Related Quality of Life
hsCRP	High sensitivity C- reactive protein
IgF	Insuline-like growth factor
IL	Interleukin
IOTF	International Obesity Task Force
ITQoL	Infant Toddler Quality of Life Questionnaire
JGZ	Jeugdgezondheidszorg
Kg	Kilograms
NICU	Neonatale intensive care unit
MUAC	Mid Upper Arm Circumference
Mg	Milligram
OLVG	Onze Lieve Vrouwe Gasthuis
PEA POD	An air displacement plethysmography system to determine body composition
PEDsQL	Pediatric Quality of Life Inventory
RCT	Randomized Controlled Trail
RDP	Rat dental pulp cells
SAE	Serious adverse event
SCORAD	SCORingAtopicDermatitis
Sd	Standard deviation

SDS	Standard Deviation Score
SF	Skin folds
SGA	Small for gestational age
SUSARs	Suspected unexpected serious adverse reactions
TAT	Thrombin-Antithrombin complex
TNF- α	Tumor Necrosis Factor-α
UK	United Kingdom
VAT	Visceral adipose tissue
vWF	von Willebrand Factor
WC	Waist Circumference
WHO	World Health Organization
WMO	Wet medisch-wetenschappelijk onderzoek met mensen

SUMMARY

Rationale: optimal timing of introduction of complementary feeding during infancy is necessary for both nutritional and developmental reasons. Limited evidence is available about the optimal age of solid food introduction in preterm infants and implications for both short and long term health.

Objective: to analyze the effect of early (12 weeks corrected age) versus late (17 weeks corrected age) introduction of complementary feeding on obesity at the age of 2 years in preterm infants.

Study design: the study comprises a randomized parallel group open-label controlled intervention study in which premature born children will be randomized to receive complementary feeding at the corrected age of 12 weeks or at the corrected age of 17 weeks.

Study population: preterm infants born between 30 and 35 $^{6}/_{7}$ weeks gestational age.

Intervention: the intervention group will start complementary feeding at 12 weeks corrected age. The control group will start complementary feeding at the corrected age of 17 weeks. The pattern and structure of the complementary feeding will be according to the advice of the well baby clinics in the Netherlands.

Main study parameter/endpoint: the main study parameter is the prevalence of obesity at the age of 2 years determined by BMI-score, according to the IOTF cut-off values and will be interpretated as association.

Nature and extent of the burden and risks associated with participation, benefit and group relatedness: the risks associated with this study are minimal. There will be no benefit for the individual participating in this study. To determine correlations between height, weight, BMI, and eating behaviour in preterm infants it is important to perform this study in this age category.

1. INTRODUCTION AND RATIONALE

1.1 Complementary feeding

Complementary feeding is defined as the introduction of non-(breast) milk foods or nutritive liquids when milk alone is no longer sufficient to meet all nutritional requirements. In this period, there is a gradual transition to eating family foods.[1] Complementary feeding is associated with major changes in both macronutrient and micronutrient intake. In healthy term-born infants living in Europe the recommendations on the age at which complementary feeding should be introduced are based on considerations for the optimal duration of exclusive breastfeeding. [2]

In 2001, the World Health Organization revised its global recommendations for the introduction of complementary feeding from 4-6 months to 6 months of age. Many countries have adopted this recommendation, whereas other countries continue to recommend the introduction of complementary feeding between 4 and 6 months.[3-6] The ESPGHAN Committee recommended in 2008 that the introduction of complementary feeding should not be before 17 weeks but should not be delayed beyond 26 weeks of age in all infants, acknowledging exclusive or full breastfeeding until 6 months as a desirable goal.[7] At about 6 months the volume of ingested human milk by exclusively breast-fed infants becomes insufficient to meet the requirements of energy, protein, iron, zinc, calcium, selenium and some fat-soluble vitamins (A and D).[2;7]

1.1.1 Complementary feeding in preterm infants: the benefits and risks

However, the above mentioned recommendations concern healthy term-born infants. These guidelines cannot be directly translated to preterm infants. Preterm infants are a heterogeneous population because their gestational age at birth could vary between 23 to 36 weeks. In the Netherlands the incidence of preterm birth (<37 weeks gestational age) is 7.7% and very preterm birth (<32 weeks gestational age) 1.3%.[8] In 2013 171,000 children were born alive in the Netherlands.[9]

Limited evidence is available about the optimal age of solid food introduction in preterm infants and implications for both short and long term health. A recent review by Palmer et al outlined the challenges of introducing solid foods to preterm infants and evaluated the benefits and risks.[10] According to this review starting solid foods in every premature infant should be individualized taking into account infants' gestational age at birth, early nutrition intake, current nutritional status and requirements, as well as developmental progress and readiness. Optimal timing of the introduction of complementary feeding during infancy is necessary for both nutritional and developmental reasons. The possible harms of early

introduction of solid foods, considering the risk of infection, maturation of renal function, and the development of allergies are discussed. [10]

Infection: Currently available evidence does not support that there is an increased infection rate. Despite their immaturity the majority of preterm infants are digesting and absorbing whole proteins, lactose and dietary fats well before they reach term due date; some many months earlier. The digestive capacity of the preterm infant starts lower than in the term born counterpart, but maturation accelerates postnatal to give adequate function by the time weaning is likely to occur.[11] In preterm infants the gut barrier appears to adapt postnatal independent of the gestational age or birth weight. During the first two days of birth the intestinal permeability in preterm infants is higher than in healthy term born infants. However, at 4-7 days of age no differences in gut permeability in preterm en term born infants are shown.[11;12]

Renal function: The majority of infants born preterm should have sufficient renal function to process increased renal solute load of nutrient-dense solid foods.[10;13;14]

Iron status: Preterm infants and especially very low birth weight infants are at risk developing iron deficiency anemia because of low iron stores at birth, rapid depletion of iron stores owing to phlebotomy losses and inability to regulate iron absorption by the gastrointestinal tract.[15] The optimal timing and duration of iron supplementation remains undetermined.[16] The American Academy of Pediatrics recommended an iron intake of at least 2 mg/kg per day through 12 months of age. Preterm infants fed human milk receive an iron supplement of 2 mg/kg per day by 1 month of age. This should be continued until the infant is weaned to iron-fortified formula or begins to eat complementary foods supplying the 2 mg/kg per day.[17] Few data is available on the relationship between complementary foods and iron status. ESPGHAN Committee on Nutrition reviewed the relationship between the specific food and iron status.[2] An increase in meat intake prevented a decrease in hemoglobin in late infancy, but had no effect on iron stores or on cellular iron deficiency in partially breast-fed infants between 8 and 10 months in a randomized trial. Engelmann et al found a little effect on iron or zinc status when weaning cereals with different phytate contents.[18] Furthermore, iron bioavailability from iron-fortified infant cereals can be improved by using an iron compound with high relative bioavailability and by ensuring adequate ascorbic acid content of the product.[36;37]In order to determine the effect of complementary food on iron status in a recent Icelandic randomized trial was examined the term infants with a small amount of complementary food in addition of breast milk from 4 months of age and exclusively breast feed infants for 6 months. A small positive effect on ferritin levels at 6 months was seen, but no effect of iron deficiency, and growth.[19] Marriott et al showed a significant improved iron status in the early weaning group (starting from 3 months) at 6 months gestational corrected age.[20]

Allergies: Lastly, there are contrasting reports on the risk of developing food allergy due to early exposure of solid foods.[10] It was thought that with early introduction of solid food the preterm infant might have a risk of developing food allergy. An observational study investigated 257 preterm infants born in the UK showed that infants who had 4 or more solid foods introduced at <17 weeks of corrected age had an increased risk (odds ratio (OR) 3.49; 95%CI 1.51-8.05) of developing eczema by 12 months of corrected age.[21] This study also reported that the introduction of solid foods prior to 10 weeks of corrected age was associated with an increased risk (OR 2.94; 95%CI 1.57-5.52) of eczema development. However, information was collected retrospectively on solid food practices (recall bias). The relation between food hypersensitivity and atopic dermatitis is controversial. A Danish Allergy Research Centre Cohort, including 562 children, found that only 15% of the children with constitutional eczema also had a food allergy.[22] More recent papers are in contradiction with the study above. Liem et al investigated whether premature or low birth weight children have an increased risk of developing food allergy compared with term or normal birth weight children from birth to seven years. [23]They studied a birth cohort of 13.980 children, of which were 881 preterm children (6.3%), in Canada. According to this study immaturity of the gastrointestinal tract or immune response does not seem to change the risk for development risk for allergies.[23;24] Kvenshagen et al found no difference in the prevalence of eczema/atopic dermatitis at 2 years of age between 32/161 (19.9%) children born preterm and 63/351 (17.9%) born at term. [24] There was also no difference in the prevalence of IgE-mediated food allergy in children with atopic dermatitis between infants born preterm (2/32, 6.2%) and term (6/63, 9.5%). According to the most recent literature it seems that preterm infants are no more likely to develop atopic disease than term infants.[11]

Development: King outlined the oro-motor development of the preterm infants and concluded that it is not necessary to wait for lip seal to develop and tong protrusion to diminish before weaning – in fact they may only mature with the aid of weaning. [11]The delay in introduction of lumpy foods can lead to feeding problems if a sensitive period is missed. The later a preterm infants is introduced to new tastes the less likely they are to accept a wide variety of foods.[11] There is also the belief that the introduction of solid foods assists speech development through an increased variety of use of tongue and jaw muscles.[10]

1.1.2 The start of complementary feeding and developing obesity

The literature is still conflicting regarding complementary feeding as a determinant of overweight and obesity. In 2012, Przyrembel gave an overview of the literature on the effect of early diet in infancy and young childhood on health outcomes in childhood/adulthood. [25] They also focused on obesity. Some longitudinal observational studies suggest that early (i.e.

age <12–17weeks), introduction of complementary food may increase the risk of overweight/obesity or body fat in child- and adulthood, compared to introduction at age >17 weeks. The risk may be smaller for breastfed than non-breastfed infants. The author strongly advised against the introduction of complementary food before the age of 12 weeks, and concluded that an introduction before the age of 17 weeks may be associated with adverse health consequences in later life and is not associated with any apparent health benefit. On the other hand delaying the introduction of complementary food beyond the age of 26 weeks is associated with the risk of nutritional insufficiency.[25] A recent systematic review in 2013 about the timing of introduction of complementary feeding and the risk of childhood obesity in term infants showed that very early introduction of solid foods (≤4 months of age) may result in an increase in childhood BMI.[26] A recent randomized controlled trial also showed no effects of exclusive breastfeeding for 4 or 6 months on the growth pattern or the risk of being overweight or obese in early childhood in term infants. [27] The follow-up was performed until 38 months of age in which weight, height and head circumference of the infants was measured. However, since this systematic review, Huh et al found that the introduction of solids before the age of 4 months was associated with a six-fold increase in the risk of obesity at age 3 years and Seach et al showed that delayed introduction of solids is associated with reduced odds of child overweight/obesity.[28;29] Furthermore, Weng et al reported in a systematic review published in 2012 that there was some evidence associating early introduction of solid foods and childhood overweight. [30] This systematic review included the papers of Huh et al and Seach et al.

Childhood obesity is associated with major health risks. There is evidence that overweight youth are at increased risk of remaining overweight. A recent review reported increased risk for overweight or obese youth to become overweight or obese in adulthood.[31] Obesity has many consequences on children's health. There are metabolic disturbances, such as insulin resistance, dyslipidemia, and hypertension. Wahl et al identified the serum metabolites associated with childhood obesity among 120 children, of which 80 were obese children, in a cross-sectional study. [32] They found 14 metabolites and 69 metabolite ratios significantly different in obese compared to normal-weight children. Furthermore, visceral adipose tissue (VAT) and adipose tissue resident macrophages produce many pro-inflammatory cytokines. [33] These cytokines can induce insulin resistance and play a major role in the pathogenesis of endothelial dysfunction and subsequent atherosclerosis.[34] These cytokines include for example Tumor Necrosis Factor-α (TNF- α), Interleukin-6 (IL-6), and less adiponectin and interleukin-10 (IL-10). Circulating inflammatory markers (TNF-α, high sensitivity C- reactive protein (hsCRP), IL-6) and hemostatic markers (fibrinogen, von Willebrand Factor (vWF), D-dimer, Thrombin-Antithrombin complex (TAT)) and leptin levels are higher in obese children in comparison with non-obese children.[35] It is suggested that information on pro- and

inflammatory markers and metabolomics in children with obesity should be taken into consideration in the hypotheses on obesity-related pathophysiological processes.

1.1.3 Current advice for the timing of complementary feeding in preterm infants

The delayed early gross motor developmental progress in preterm infants should be taken into account while determining the timing of the introduction of complementary feeding. This developmental milestone may be delayed up to 3 months of corrected age.[10] Good head control is the important developmental milestone for safe starting of solid foods.[36] The ESPGHAN committee advised that if their baby has a reduced tongue trust (protrusion) reflex, can sit in a stable supported position, can hold its head up well, opens its mouth, and leans forward towards the spoon the baby is developmentally ready for solid foods.[10] These recommendations were based on the limited studies that are available in preterm infants assessing the practices of complementary foods. The only guideline relevant for introducing solid foods to preterm infants was the advice from the Department of Health in the United Kingdom (1994).[37] This guideline recommends introducing solid foods when the preterm infant reaches a weight of 5 kg, has lost the extrusion reflex, and is able to eat from a spoon. However, it does not include an age recommendation. Observational studies in developed countries have found that solid foods have been introduced to the majority of the preterm infants prior to 4 months of corrected age.[38-41] King has suggested that most preterm infants may be ready for solid foods between 5 and 8 months of uncorrected age, provided that the infant is at least 3 months of corrected age (gross motor development should enable safe eating).[11] Palmer et al recommended that 3 months (13 weeks of corrected age) are considered as an appropriate age to start high protein, energy and nutrient rich solid foods for otherwise health preterm infants.[10] Considering the corrected age the earliest born infants at 23 weeks of gestation would have an uncorrected age of 7 month, while the late preterm infants would have an uncorrected age of 4 months.

In conclusion, both early as late start of complementary feeding could have adverse consequences. Optimal timing of the introduction of complementary feeding during infancy is necessary for both nutritional and developmental reasons. Most preterm infants may be ready for solid foods between 5 and 8 months of uncorrected age, provided that the infant is at least 3 months of corrected age. Gross motor development should enable safe eating. Hence, taking into account all the above described factors, there is insufficient or contrasting information about the effect of the timing of complementary feeding in preterm infants. Moreover, information about the timing of complementary feeding and obesity in preterm

infants is missing. Given these contrasting reports on the introduction of solid foods in premature born infants, randomized controlled trials (specifically designed to assess the risks and benefits of the timing of introduction of solid foods) are needed. Further research is required to provide evidence based guidelines specifically for preterm infants and studies should investigate immediate as well as long-term consequences of the pattern and timing of introducing solid foods on later health and developmental outcomes. The number of overweight or obese children is increasing. A recent review found strong evidence of overweight tracking into adulthood, so if the number of overweight children is increasing, then so will the number of overweight adults. The expectation is, therefore, that preventing excess weight gain in childhood will help to reduce adult overweight and obesity. [26;31] Therefore, the main objective of this study is to analyze the effect of early introduction versus late introduction of complementary food in preterm infants on the prevalence of obesity at the age of 2 years.

2. OBJECTIVES

Main objective:

The main objective of this study is to analyze the effect of early (12 weeks corrected age) versus late (17 weeks corrected age) introduction of complementary food (weaning) on the prevalence of obesity at the age of 2 years in preterm infants. Overweight (including obesity) is determined by measuring the BMI, according to the IOTF cut-off values.[42] Preterm infants are included when born between 30 and $35\,{}^{6}/_{7}$ weeks of gestational age.

The difference in weeks between the early and late group seems to be small. Palmer et al recommended that 13 weeks is considered as an appropriate age to start with complementary feeding and King suggested the infants should be at least 3 months corrected age, meaning 12 weeks (see table 1).[10;11] For this reason, the early intervention group will start with complementary feeding at 12 weeks corrected age. However, starting with complementary feeding at 17 weeks corrected age in a child born at 30 weeks gestational age means that the uncorrected age or postpartum age is 27 weeks. Starting with complementary feeding at the uncorrected age of 27 weeks corresponds with approximately 6.2 months (see table 1). This is the latest acceptable time to start with complementary feeding, since research has proven that at about 6 months the volume of ingested human milk by exclusively breast-fed infants becomes insufficient to meet the requirements of energy, protein, iron, zinc, calcium, selenium and some fat-soluble vitamins (A and D).[7] [2] In conclusion, these considerations leaded to the early group (12 weeks corrected age) and late group (17 weeks corrected age) to start with complementary feeding.

GA	Corrected age	Postpartum age
30	Early: 12 weeks	22 weeks
$35\,{}^{6}/_{7}$	Late: 17 weeks	21 weeks
30	Late: 17 weeks	**27 weeks**
$35\,{}^{6}/_{7}$	Early: **12 weeks**	16 weeks

Table 1: Showing the post-partum age of the intervention groups. GA = gestational age. In bold: 12 weeks is corresponding with 3 months corrected age, 27 weeks is corresponding with 6.2 months uncorrected age (= postpartum age).

Sub-objectives are:

1. Overweight (including obesity): to determine the effect of early versus late introduction of complementary feed on the BMI, according to the IOTF cut-off values at 1 year.
2. Eating behavior and tolerating complementary feeding: to determine if early introduction is correlated with less eating problems in comparison to introduction the late introduction, by using the Baby Eating Behaviour Questionnaire (BEBQ) and Child Feeding Questionnaire (CFQ).
    a. Subgroup analysis will be performed to explore if children with respiratory problems after birth requiring intubation have more eating problems than children without respiratory problems requiring intubation
    b. Subgroup analysis will be performed to study if the duration of a feeding tube after birth is correlated with eating problems
    c. Subgroup analysis will be performed to study if gestational age is related to eating disorders.
3. Eating behavior and tolerating complementary feeding: to determine if term born infants (control group B) have less eating problems in comparison to preterm infants before the start of complementary feeding, by using the Baby Eating Behaviour Questionnaire (BEBQ)
4. Growth parameters (weight, length, head circumference, BMI, weight-for-age, length-for-age, head circumference-for-age): comparing the growth parameters of preterm infants with term infants before the start of complementary feeding, at 1 year and at 2 years
5. Taken into account the food diaries at 6 months, 9 months, 12 monhts and 24 months and the start of complementary feeding
6. Health Related Quality of life (HRQoL): to evaluate the difference in HRQoL between early versus late introduction of complementary feeding, by using The Infant Toddler Quality of Life (ITQoL) Questionnaire or Pediatric Quality of Life Inventory (PedsQL).
7. Ages and Stages Questionnaire (ASQ): to evaluate the difference in development between early versus late introcution of complementary feeding, by using the ASQ at the age of 1 and 2 years.
8. Allergy/Atopic: to evaluate if the incidence of allergies is different between early versus late introduction of complementary feeding, by using the SCORAD.
9. Microbiota: to evaluate if the microbiota change due to the introduction of complementary feeding

:

All sub-objectives will be measured at the corrected age. For the main objectives as well as the sub-objectives sub-analysis will be performed for different categories of gestational age. These categories include gestational age before 32 weeks versus gestational age after 32 weeks.

3. STUDY DESIGN

The study comprises a multicenter randomized parallel group open-label controlled intervention study in which premature born children will be randomized to receive complementary feeding at the corrected age of 12 weeks (intervention group) or at the corrected age of 17 weeks (control group). The pattern and structure of the complementary feeding will be according to the advice of the well baby clinics in the Netherlands, using a standardized nutrition schedule.

Stratification will take place according to center.[43] In order to keep the number of subjects in the different groups closely balanced at all times block randomisation will be used. According to Altman the block size used is a multiple of the number of treatments.[9;44] A blocked randomization schedule, using blocks of four, will be used. The randomisation will be computer generated by Castor edc. The research is funded by Nutricia Nederland. Nutricia Nederland will have no financial interest in this study, will not delivery food products, will not have veto in how publication of results will be. In case of any disagreement regarding publication of the data, the principal investigators (HvG, EF, AJ and KV) of the research will make the final decision. All data analysis will be supervised by the principal investigators.

Hospital visits for all preterm infants

The participants will be seen in the hospital before the start of complementary feeding, at corrected age1 year and 2 years.

1. ***Hospital visit before the start of complementary feeding***
    a. Information about the randomisation will be given. Parents receive a letter in which is stated when to start with complementary feeding
    b. Weight, height, head circumference, and SCORAD score will be measured
    c. A diary regarding the breast- or formula milk is taken
    d. The Baby Eating Behavior Questionnaire is taken
2. ***Hospital visit at corrected age 1 year***
    a. Weight, height, head circumference, and SCORAD score will be measured
3. ***Hospital visit a corrected age 2 years***

a. Weight, height, head circumference, and SCORAD score will be measured
b. Infant Toddler Quality of Life Questionnaire is taken
c. Child Feeding Questionnaire is taken
d. Ages and Stages Questionnaire is taken

All hospital visits will be combined with regular hospital visits if possible. We expect the preterm infants to have a regular hospital visit before the starting of complementary feeding and at 1 year. The hospital visit at the age of 2 years will be an extra hospital visit. In this study, measurements recorded for standard care and extra measurements taken as part of this study are used.

Standard measurements during hospital visit:

- Weight, height, head circumference

Extra measurements:

- SCORAD (**SCOR**ing**A**topic**D**ermatitis, a clinical tool used to assess the extent and severity of eczema).
- The eating behaviour (Baby Eating Behaviour Questionnaire (BEBQ)) is measured before the start of complementary feeding.
- Infant Toddler Quality of Life Questionnaire (ITQoL) or Pediatric Quality of Life Inventory (PedsQL) is measured at corrected age of 2 years.
- The Child Feeding Questionnaire (CFQ) will be measured at corrected age of 2 years.
- The Ages and Stages Questionnaire (ASQ) will be measured at corrected age of 1 and 2 years.
- Diary: Parents of all children are asked to complete three different diaries during the study period until age of 2 year.
  - Version 1 (Milk diary): A diary about the breast- or formula milk before the start of complementary feeding.
  - Version 2 (Start diary): A diary at the start of complementary feeding. Parents are asked to fill in when they give solid food for the first time, what kind of solid food they give and how much their child eats for the first time.
  - Version 3 (2 day diary): A 2-day diary method: Parents will be asked to fill in the food diary during two days, of which one day in the weekend. Parents are asked to do this at the age of 6 months, 9 months 1 year and 2 years.
- Microbiota: parents of all preterm children are asked to collect faeces at home at three different time points:
  - 10-11 weeks post term
  - 15-16 weeks post term

- <u>20-21 weeks post term</u>

If the partens give informed consent for collecting faeces, the preterm infants are part of group 2a. We will include a total of 150 patients for microbiota ananalysis. In case parents wish to attent the study without collecting faeces, they will be part of group 1 (see figure 1 and 2)

The measurements are collected at the hospital. If this is not possible, height and weight at the age of one and two years will be collected at the well baby clinics (with permission of the parents). The reach of the well baby clinics, part of the Child Health and Welfare service (JGZ), in the Netherlands is high. Ninety-eight percent of all the newborns and 95% of all the infants attend the well baby clinics.[45] Regular visiting moments of the well baby clinics are given in table 2. The well baby clinics and the general practitioner will be informed about the study and the group in which the patients are allocated.

**Age**	**Kind of contact**
4th -7th day	Home visit
2th week	Home visit
4th week	Visit well baby clinic
8th week	Visit well baby clinic
3 months	Visit well baby clinic
4 month	Visit well baby clinic
6 month	Visit well baby clinic
7,5 months	Visit well baby clinic
9 months	Visit well baby clinic
11 months	Visit well baby clinic
14 months	Visit well baby clinic
18 months	Visit well baby clinic
2 year	Visit well baby clinic
3 year	Visit well baby clinic
3 years 9 months	Visit well baby clinic

Table 2: Regular contact moments well baby clinics for children 0-4 years old in the Netherlands.[46]

This study is registered at Nederlandse Trial Register (TC = 4939) and is being registered at clinical trials.gov.

Measurements for control group B (term born children)

The term born infants will be enrolled in two hospitals, or obstetric practices and well baby clinics affiliated with the hospital, in which the preterm infants are also enrolled. The parents of the term infants will be informed with a brochure by the midwive, nurse or medical doctor during a obstetric practice visit, outpatient clinic/well baby visit or during the hospital stay/visit for the delivery. If parents give approval to the midwive or doctor, the midwive or doctor will inform the researcher or research nurse. They will contact parents by phone and provide additional information and give or send the information letter and informed consent form to the parents. Parents will be give at least one week to consider their decision after receiving the information letter and informed consent form. Informed consent will be taken by the researcher or research nurse. Treatment or follow-up of the term born child will not change depending on participation in this study. Parents will be given at least one week to consider the information before making the decision on participation. If there are no further questions, informed consent will be taken.

Parents of the term born infants are asked to:

- Fill in the BEBQ and a questionnaire regarding parental characteristics (see 8.1.3) when their child is 3 months old
- Send information to the researchers about the growth parameters (length, weight, head circumference) of their child at 3 months, 1 year and 2 years old, obtained at the well baby clinic
- Fill in four food diaries when their child is 6 monhts, 9 months, 12 months and 24 months old

4. STUDY POPULATION

4.1 Population (base)

Preterm infants born between 30 and 35$^6/_7$weeks gestational age

4.1.1 Inclusion criteria

- Preterm infants born between 30 and 35$^6/_7$weeks gestational age
- Informed consent from both parents

4.1.2 Exclusion criteria

- Small for Gestational Age (SGA) (<p2.3)
- Diseases interfering with stable growth
- Intestinal disorders (necrotizing enterocolitis needing surgery, short bowel syndrome, hirschsprung disease, inflammatory bowel disease)

- Moderate and severe bronchopulmonary dysplasia (BPD) defined according to the international criteria[47]
- Kidney disorders
- Congenital heart disease with hemodynamic consequences
- Severe cow milk allergy (Samson score $\geq$ 3)
- Congenital anomalies Ear Nose Troat (ENT) area, esophageal and or tracheal, needing operative correction (e.g. cheilognatopalatoschizes, esophageal atresia)
- Syndromal disorders (e.g. trisomie 21, PraderWilli)
- Intra ventricular hemorrhage grade III or IV
- No motivation of parents
- No informed consent

4.2 Control population

The control population exists of preterm infants born between 30 and $35^{6}/_{7}$weeks gestational age. These children will start with complementary feeding at the corrected age of 17 weeks. The structure of complementary feeding will be the same as the children who start with complementary feeding at the corrected age of 12 weeks.

Control group B:

Control group B exists of term born infants.

The inclusion criteria for the term born children are:

- Term born infant (born between 37 and 42 weeks of gestational age)
- Written informed consent from both parents, or legal representive

The exclusion critera for the term born children are:

- Small for Gestational Age (SGA) (<p2.3)
- Diseases interfering with stable growth
- Intestinal disorders (necrotizing enterocolitis needing surgery, short bowel syndrome, hirschsprung disease, inflammatory bowel disease)
- Moderate and severe bronchopulmonary dysplasia (BPD) defined according to the international criteria[47]
- Kidney disorders
- Congenital heart disease with hemodynamic consequences
- Severe cow milk allergy (Samson score $\geq$ 3)

- Congenital anomalies Ear Nose Troat (ENT) area, esophageal and or tracheal, needing operative correction (e.g. cheilognatopalatoschizes, esophageal atresia)
- Syndromal disorders (e.g. trisomie 21, PraderWilli)
- Intra ventricular hemorrhage grade III or IV
- No motivation of parents
- No informed consent

4.3 Sample size calculation

The sample size calculation for this study is based on previous reported effects. In a population based study Jingxiong et al reported that introduction of complementary feeding before the age of 4 months resulted in a 76% higher odds in developing overweight than children introduced to complementary feeding after 4 months.[48] Overweight in this study was defined as weight-for-length/height ≥ 2SD above the median of the WHO reference for sex, i.e. weight-for-length/height z-score ≥ 2SD. A study by Young et al reported that excess weight gain (defined as gaining ≥0.5 in weight-for-age Z-score) between 4 to 6 months was associated with an odds ratio of 2.5 for overweight at 18-24 months[49].This leaded to the conclusion that rapid early weight gain, for example due to early weaning, could result in obesity since known post-natal risk factors for obesity are rapid early weight gain and early weaning.[50] Based on these studies we expect that the introduction of early complementary feeding in children will result in a higher prevalence of overweight children at the age of 2 year in comparison to later introduction of complementary feeding. Based on the given odds ratio of 1.76 and 2.5 an odds ratio of 2.3 was used to calculate our sample size. In 2009, the prevalence of overweight was 8% in two year old children in the Netherlands. Overweight was determined by measuring the BMI. BMI was calculated as weight/height^2 and expressed as kg/m^2. Overweight and obesity prevalence rates were calculated using International Obesity Task Forse (IOTF) cut-of values. In this study, the same calculation and definition will be used.[51]Information of the prevalence of overweight in premature infants is lacking. Bocca-Tjeertes et al has shown that preterm infants are a growth restraint compared to term infants.[52] On the other hand, Euser et al found a greater fat mass normalized for weight in preterm infants.[53] Given this contrasting information on preterm infants, a slightly lower prevalence of overweight of 6% was used to calculate the sample size. Using the odds ratio of 2.3, the intervention group will have a prevalence of overweight of 13%. With a 0.05 significance level and a power of 80% a minimal sample size of 275 patients per group is required to detect a significant increase in the prevalence of overweight. Loss to follow-up is

calculated to be 5%. This is based on the fact that 95% of all the infants attend the well baby clinics in the Netherlands.[45] In case patients will not attend the hospital visits, the well baby clinics will be contacted for information about height and weight. This means that 579 children have to be included in the study, finalized by a number of 600 children.

5. TREATMENT OF SUBJECTS

5.5 Investigational product/treatment

The intervention group will start complementary feeding at the corrected age of 12 weeks, when gross motor development should enable safe eating.[11] The control group will start complementary feeding at the corrected age of 17 weeks. The pattern and structure of the complementary feeding will be according to the advice of the well baby clinics in the Netherlands, using a standardized nutrition schedule.

The parents will receive information direct after the randomization about the group allocation. One week before they are assigned to start with complementary feeding, they will receive a reminder by phone. If this is not possible an email or a letter by post will be send. Parents will have one week to start with complementary feeding, either at the corrected age of 12 weeks or at the corrected age of 17 weeks. Parents will receive detailed information about complementary feeding, what kind of food to start with, how often they have to give solid foods, and some recipes. The introduction of complementary feeding is divided in 4 steps: 1) introduction of vegetables, 2) introduction of fruits, 3) introduction of cereal, and 4) introduction of a warm meal. Parents receive information when to move on to the next step. The well baby clinics and attending pediatricians are informed about the group allocation and are asked to support parents to start with complementary feeding by the time they are allocated. They also receive the information that the parents received regarding how to start with complementary feeding. In case there is a reason not starting complementary feeding at the time of allocation, parents are asked to report this and the reason why not starting in the diaries. Furthermore, the diaries will point out at what time the parents started with complementary feeding.

For the term born children (control group B) there will be no intervention with complementary feeding.

5.6 Use of co-intervention (if applicable)

The use of co-medication is allowed in all patients.

5.7 Escape medication (if applicable)

Not applicable

6. INVESTIGATIONAL PRODUCT

Not applicable.

6.1 Name and description of investigational product(s)

6.2 Summary of findings from non-clinical studies

<One may refer to the Investigator’s Brochure (IB), Investigational Medicinal Product Dossier (IMPD), Summary of Product Characteristics (SPC) or a similar document (if applicable), by mentioning the relevant pages in that document. Be sure that the information is up to date and references to peer reviewed papers in (biomedical/scientific) journals should be given where appropriate.>

6.3 Summary of findings from clinical studies

<See explanatory text of chapter 6.2, including remark>

6.4 Summary of known and potential risks and benefits

<See explanatory text of chapter 6.2, including remark>

6.5 Description and justification of route of administration and dosage

6.6 Dosages, dosage modifications and method of administration

6.7 Preparation and labelling of Investigational Medicinal Product

6.8 Drug accountability

<Please describe the procedures *for the shipment, receipt, disposition, return and destruction of the investigational medicinal products.>*

7. NON-INVESTIGATIONAL PRODUCT

Not applicable, see chapters 7.1; 7,6; and 7.7.

<This chapter is applicable for any other product that is used in the study, like challenge agents or products used to asses end-points in the trial. This can be a medicinal product or a food product or a chemical compound or stable isotope or other product.
This chapter does ***not*** *include co-medication or escape medication, these are already mentioned in chapter 5*

For products to be used as in usual clinical practice the information can be limited to the chapters 7.1, 7.6 and 7.7 >

7.1 Name and description of non-investigational product(s)

7.2 Summary of findings from non-clinical studies

<One may refer to the Investigator's Brochure (IB), Investigational Medicinal Product Dossier (IMPD), Summary of Product Characteristics (SPC) or a similar document (if applicable), by mentioning the relevant pages in that document. Be sure that the information is up to date and references to peer reviewed papers in (biomedical/scientific) journals should be given where appropriate.>

7.3 Summary of findings from clinical studies

<See explanatory text of chapter 7.2, including remark>

7.4 Summary of known and potential risks and benefits

<See explanatory text of chapter 7.2, including remark>

7.5 Description and justification of route of administration and dosage

7.6 Dosages, dosage modifications and method of administration

7.7 Preparation and labelling of Non Investigational Medicinal Product

7.8 Drug accountability

<Please describe the procedures for the shipment, receipt, disposition, return and destruction of the non-investigational medicinal products.>

8. METHODS

8.1 Study parameters/endpoints

8.1.1 Main study parameter/endpoint

The main study parameter of this study is the prevalence of obesity determined by measuring the BMI at the corrected age of 2 years, according to the IOTF cut-off value.[42] In addition, BMI will also be determined before the start of complementary feeding and at the corrected age of 1 year. A variation of 6 weeks before and after the corrected age of 1 and 2 years will be accepted.

8.1.2 Secondary study parameters/endpoints

The secondary study parameters:

- Eating behavior of the child by using and tolerating complementary feeding, by using the BEBQ and CFQ.
- Health Related Quality of life by using the ITQoL of or Pediatric Quality of Life Inventory (PedsQL).
- Development by using the Ages and Stages Questionnaire (ASQ)
- Allergies, by using the SCORAD.
- Microbiota of faeces

8.1.3 Other study parameters (if applicable)

Other study parameters are age, gender, height, weight status, family history, allergy/atopy status, education, social economic status, and ethnicity of the parents. Maternal information regarding the pregnancy, parity and delivery are collected as well, including maternal illness during pregnancy, such as maternal diabetes mellitus, hypertensive disorders during pregnancy, and thyroid disease. This parameters will be obtained via digital questionnaires or child records. Information on perinatal and early life factors, such as APGAR-score, birth weight, reason for prematurity, respiratory support, blood transfusion, antibiotics use, NICU admission, type of feeding until complementary feeding is started, and illness are prospectively collected. Furthermore, information on sickness and medical check-ups.

8.1.4 Study parameters tested in controls

The control group will be the group starting with complementary feeding at the corrected age of 17 weeks. All parameters will be tested in the intervention group and in the control group.

Control group B:

- Growth parameters: Length,weight and head circumference at 3 months (with an age range of 2 weeks), and 1 year and 2 years of age (with an age range of 6 weeks).
- Other parameters (see 8.1.3)
- BEBQ (questionnaire) at 10-12 weeks of age, supplemented with questions regarding the baby feeding
- Food diaries at 6 months, 9 months, 1 year and 2 years of age, supplemented with questions about the start of complementary feeding (retrospective)

8.2 Randomisation, blinding and treatment allocation

Stratification will take place according to center.[43] In order to keep the number of subjects in the different groups closely balanced at all times block randomisation will be used. According to Altman the block size used is a multiple of the number of treatments.[44] A blocked randomization schedule (randomly permuting blocks of four and six) at a time will be used. The randomisation will be computer generated by Castor edc. Twins will be randomized as well; the first twin will be randomized into one of the two groups, the second twin will be assigned to the same group. Patients will be randomised to introduction of complementary feeding at the corrected age of 12 weeks (intervention group) and to introduction of complementary feeding at the corrected age of 17 weeks (control group) (1:1). The allocation of the timing of the start of treatment will not be blinded.

8.3 Study procedures

8.3.1 **Height** – Standard care

Height will be measured in centimetre (cm). Height will be measured recumbent in centimetres in children unable to stand. Whenever the child can stand height will be measured using a stadiometer standing (without shoes) in centimetres (cm).

8.3.2 **Weight** – Standard care

Weight will be measured in kilograms (kg) in underwear without diaper.

8.3.3 **Body Mass Index (BMI)** – Standard care

BMI will be calculated by the following formula. BMI = Weight (kg) / height $(m)^2$. The Standard Deviation Score (SDS) of the Dutch growth standards will be calculated.

Overweight and obesity at the age of 2 year will be determined using the IOTF criteria. [42] The participants will be compared to the Dutch population.

8.3.4 SCORAD Index – Extra in this study

The SCORAD Index is a clinical tool used to assess the extent and severity of eczema. To determine extent, the sites of affected eczema are shaded on a drawing body. The affected area is calculated as a percentage of the whole body. In addition a representative area of eczema is selected. In this area the intensity of the signs redness, swelling, oozing/crusting, scratch marks, skin thickening (lichenification) and dryness each is assessed as none (0), mild (1), moderate (2) or severe (3). Subjective symptoms (i.e. itching, sleeplessness) are scored by the caregiver using a visual analogue scale. Zero means no itch/no sleeplessness and 10 is the worst imaginable itch (or sleeplessness).[54] The SCORAD Index will be used during all the hospital visits; before the start of complementary feeding, at 1 and 2 years of age.

8.3.5 Health Related Quality of Life (HRQoL): Infant Toddler Quality of Life Questionnaire (ITQoL) or Pediatric Quality of Life Inventory (PedsQL) – Extra in this study

HRQoL is a multidimensional concept covering different domains. These domains are physical, occupational, psychological and social functioning. Generic measures summarize a spectrum of domains and dimensions of health that apply equally and broadly to diverse conditions or populations.[55] HRQoL in children will be measured using the Infant Toddler Quality of Life Questionnaire (ITQoL) or Pediatric Quality of Life Inventory (PedsQL).

The ITQoL was developed for use infants and toddlers from the age of 2 months to 5 years of age. This questionnaire has 97 items and measures 12 concepts and has to be completed by the parents. It is a generic HRQoL questionnaire. Per concept the items can be summed up and transformed into a 0 (worst possible score) to 100 (best possible score) scale. This questionnaire is used in different samples of Dutch children.[56-59] The ITQoL is a generic HRQoL.

The PedsQL™ Measurement Model is a modular approach to measuring health-related quality of life (HRQOL) in healthy children and adolescents and those with acute and chronic health conditions. The PedsQL™ Measurement Model integrates seamlessly both generic core scales and disease-specific modules into one measurement system. The items of the four scales (Physical Functioning, Emotional Functioning, Social Functioning, and School Functioning) are grouped together on the actual questionnaire. Items are reversed scored and linearly transformed to a 0-

100 scale, so that higher scores indicate better HRQOL (Health-Related Quality of Life).

The parents will be asked to act as a proxy for their child and complete the questionnaire. The ITQoL or PedsQL will be measured at the age of 2 years. This questionnaire will be sent to the parents via Castor edc and will be filled out digitally.

8.3.6 Eating behavior – Extra in this study

The Baby Eating Behaviour Questionnaire (BEBQ) will be measured before the start of complementary feeding (12 weeks or 17 weeks) in both preterm and term born children (control group B). This questionnaire will be sent to the parents via Castor edc and will be filled out digitally. The BEBQ is a parent-report measure of appetite during the milk-feeding phase of infancy. Four dimensions emerged, with 17 items tapping four distinct feeding traits and one item describing general appetite. 'Enjoyment of food' (4 items) related to the infant's perceived liking of milk and of feeding in general, 'food responsiveness' (6 items) evaluates how demanding the infant is with regard to being fed and his or her level of responsiveness to cues of milk and feeding, 'slowness in eating' (4 items) measures the speed with which an infant typically feeds, and 'satiety responsiveness' (3 items) assesses how easily the infant gets full during a feed. BEBQ has shown a good reliability, with a Conbach' salfa between 0.73-0.81.

8.3.7 Child Feeding Questionnaire (CFQ) – Extra in this study

The Child Feeding Questionnaire (CFQ) will be measured at the age of 2 years. This questionnaire will be sent to the parents via Castor edc and will be filled out digitally. It is a self-report measure to assess parental beliefs, attitudes, and practices regarding child feeding, with a focus on obesity proneness in children. The current version of the CFQ contains seven factors that fall into two categories. The first category, risk factors and concerns, measures factors that may elicit parental control in feeding and contains: perceived Feeding Responsibility, Perceived Parent Overweight, Perceived Child Overweight, and Concern about Child Overweight. The second category, control in child feeding: attitudes and practices, measures parents' attitudes and behaviors regarding child feeding and contains: Restriction, Pressure To Eat, and Monitoring. The reliability of both parents and child version is good: the Cronbach's alpha is respectively >0.70 and 0.66-0.88.

8.3.8 Ages and Stages Questionnaire (ASQ) – Extra in this study

The Dutch version on the Ages and Stages Questionnaire extended version, third edition, (ASQ) will be measured at the age of 1 year and 2 years corrected age.[60] This questionnaire will be sent to the parent digitally or will be filled in on paper. The ASQ is a validated parent-completed developmental screening tool that covers 5 developmental domains: communication, fine motor function, gross motor function, personal-social functioning, and problem solving. Each domain has 8 or 9 questions on developmental milestones. Parents evaluate whether the child has achieved a milestone (yes, 10 points), has partly achieved the milestone (sometimes, 5 points), or has not yet achieved the milestone (no, 0 points). ASQ total score is calculated by adding all the domain scores and dividing the total by 5. Parents will be sent the "extended ASQ version" so for 12 months the combined questions for 12 and 14 months and for 24 months the combined questions for 24 and 27 months to calculate both a score based on chronological age and a score corrected for prematurity to improve sensitivity. The time window for the ASQ will be one month around the first and two months around the second birthday (11 to 13 months and 22 to 26 months respectively).

8.3.9 Microbiota – Collecting faeces – Extra in this study

Parents of all preterm children in group 2a are asked to collect faeces at home at three different time points:

- First sample: 10-11 weeks post term (before the start of complementary feeding)
- Second sample: 15-16 weeks post term (after the 'early-group' has started complementary feeding)
- Third sample: 20-21 weeks post term (after both 'early-group' and 'late-group' have started complementary feeding)

To evaluate if the microbiota change due to the start of complementary feeding, samples before and after the start of complementary feeding will be compared. The late group will function as a 'control group' when analyzing the second sample, since the 'early-group' has already started with complementary feeding, whereas the 'late-group' has not jet started with complementary feeding.
Parents are asked to collect three samples of the faeces at home by using a fecal swab. The fecal swab is inserted in the stool in the diaper, and afterwards the fecal swab is transferred into a tube.

8.3.10 Dietary intake – Extra in this study

Parents of all children (preterm infants and term born infansts (control group B)) are asked to complete three different diaries during the study period until the age of 2 years. The diaries will be pilot tested. Possibly, the diaries will be collected via a digital website.

- Version 1 (Milk diary): A diary about the breast- or formula milk before the start of complementary feeding.
- Version 2 (Start diary): A diary at the start of complementary feeding. Parents are asked to fill in when they give solid food for the first time, what kind of solid food they give and how much their child eats for the first time.
- Version 3 (2 day diary): A 2-day diary method: Parents will be asked to fill in the food diary during two days, of which one day in the weekend. Parents are asked to do this at the age of 6 months, 9 months, 1 year and 2 years.

8.4 Withdrawal of individual subjects

Subjects can leave the study at any time for any reason if they wish to do so without consequences. The investigator can decide to withdraw a subject from the study for urgent medical reasons.

If the parent(s) of the subject or the investigator have the idea that the subject doesn't want to participate (anymore) in this research project the subject will leave the study.

8.5 Replacement of individual subjects after withdrawal

There will be no replacement of individual subjects after withdrawal.

8.6 Follow-up of subjects withdrawn from treatment

There will be no follow-up of subjects withdrawn from the study. Subjects withdrawn from the study still participate in the regular follow-up scheme for prematurity offered by the treating hospital.

8.7 Premature termination of the study

The study will end prematurely if anything occurs, on the basis of which it appears that the disadvantages of participation may be significantly greater than was foreseen in the research proposal.

Procedures in case the study will be put on hold:

- Inclusion of new patients will stop immediately.

- All participating pediatricians will receive a letter with information about the temporarily termination of the study as well as the reason for it. They will also receive the written information for their included patients.

9. SAFETY REPORTING

9.1 Section 10 WMO event

In accordance to section 10, subsection 1, of the WMO, the investigator will inform the subjects and the reviewing accredited METC if anything occurs, on the basis of which it appears that the disadvantages of participation may be significantly greater than was foreseen in the research proposal. The study will be suspended pending further review by the accredited METC, except insofar as suspension would jeopardise the subjects' health. The investigator will take care that all subjects are kept informed.

9.2 Adverse and serious adverse events

Adverse events are defined as any undesirable experience occurring to a subject during a clinical trial, whether or not considered related to the study. All adverse events reported spontaneously by the subject or observed by the investigator or his staff will be recorded.

A serious adverse event is any untoward medical occurrence or effect that at any dose results in death;

- is life threatening (at the time of the event);
- requires hospitalisation or prolongation of existing inpatients' hospitalisation;
- results in persistent or significant disability or incapacity;
- is a new event of the trial likely to affect the safety of the subjects.

All SAEs will be reported to the accredited METC that approved the protocol, according to the requirements of that METC.

Suspected unexpected serious adverse reactions (SUSARs) are defined as an adverse reaction that is both unexpected and also meets the definition of a serious adverse event (SAE). It is classified as unexpected if its nature and severity are not consistent with the product information. Since this randomized controlled trial does not use any form of drug or test product the risk of a SUSAR is very unlikely.

9.3 Follow-up of adverse events

All adverse events will be followed until they have abated, or until a stable situation has been reached. Depending on the event, follow up may require additional tests or medical procedures as indicated, and/or referral to the general physician or a medical specialist.

9.4 Data Safety Monitoring Board (DSMB)

A DSMB is not needed for the SPOON-study, but is deemed to be appropriate. The composition of the committee and independent members are as follows:

- Clinical expert: dr. K.F.M. Joosten, working at Erasmus MC, Sophia, no conflict of interest with the sponsor
- Statisticus: prof. dr. A.H. Zwinderman, working at AMC Amsterdam, biostatistics, no conflict of interest with the sponsor
- Member of the “Vereniging van Ouders van Couveusekinderen” no conflict of interest with the sponsor

The reason for extablishing this committee is to surveillance the ongoing safety and to perform interim analyses.
The interim analyses are planned as followed:

- When the primary outcome of 50 children is collected
- When the primary outcome of 100 children is collected
- When the primary outcome of 300 children is collected

The DSMB will assess data integrity and adverse events once every year, starting after randomization of 100 patients. The DSMB will advise the investigators if the study needs to be modified or prematurely terminated.

10. STATISTICAL ANALYSIS

Since the sample size was not archived, the data will be interpretated with caution. Data will be analyzed on an intention to treat basis. In addition, we will also perform a per protocol analysis, taking into account the attrition rate. In case of missing values we will use multiple imputation. For the per protocol analysis, the criteria for the start of complementary feeding will be 12-13 weeks for the early group and 16-17 weeks for the late group. A p-value of 5% will be regarded as statistically significant and results will be presented with 95% confidence intervals.

Categorical data will be presented in absolute numbers and percentages. If a metric variable is normally distributed results will be shown in mean and standard deviation (sd). If a metric variable is not normally distributed median will be used with the interquartile range.
Descriptive data on BMI will be compared with the most recent Dutch growth chart. For the primary study parameter, the prevalence of obesity, the percentage of patients that develop overweight by using the IOTF will be calculated and intervention group and control will be compared including prevalence ratios with 95% confidence intervals. Furthermore, BMI and BMI z-scores between the intervention and control group will be compared as continuous variables.
The results of the questionnaires (BEBQ, CFQ, ITQoL / PedsQL, ASQ) will be analysed according to the scoring tables attached to the questionnaires. Allergy will be scored by a SCORAD form and can be presented as continuous or categorical variable. All blood and faeces results will be analyses as continuous or categorical variable. The diary results will be analyses as a continuous variable.
For all sub-objectives (as formulated in chapter 2), the intervention group and control group will be compared to determine the effect of early versus late introduction of complementary food, using a independent t-test or nonparametric test. For subgroup analysis, i.e. for children with respiratory problems or tube feeding, logistic regression will be used.
For control group B, the preterm infants will be compared with the term infants to determine the difference of growth parameters, BEBQ results and food diaries before the start of complementary feeding, at 1 year and at 2 years of age. Growth parameters and BEBQ will be presented as continue parameters (mean and sd or median with interquartal range). The growth parameters will be compard with the most recent Dutch growth curves. For the analyses, a t-test or nonparametric test will be used.
To take into account repeated measurement on the infants (non)linear mixed models will be used with infant as random factor and treatment (intervention, control), time and

time*treatment as fixed factors. Infant and hospital will be added as random intercept. Random slopes will be added for time dependent variables.

11. ETHICAL CONSIDERATIONS

11.1 Regulation statement

This study will be conducted according to the principles of the Declaration of Helsinki and in accordance with the Medical Research Involving Human Subjects Act (WMO) [67].

11.2 Recruitment and consent

The supervising doctor will inform the parents orally about the study and will hand over the information brochure. Parents will be give at least 24 hour to consider their decision after receiving the information brochure. If parents give approval to the supervising doctor, the investigator, supervison doctor, or research nurse will ask informed consent.This research nurse is trained and instructed by the investigator. Treatment of the child (participation in the follow-up program for prematurity in the outdoor clinic) will not change depending on participation in this study.

For the control group of term born children, the midwive or doctor will inform parents after birth (at home or in the hospital) by giving them an information brochure. If parents give approval to the midwive or doctor, the midwive or doctor will inform the researcher or research nurse. They will contact parents by phone and provide additional information and give or send the information letter and informed consent form to the parents. Parents will be give at least 24 hour to consider their decision after receiving the information letter and informed consent form. Informed consent will be taken by the researcher or research nurse. Treatment or follow-up of the term born child will not change depending on participation in this study.

11.3 Objection by minors or incapacitated subjects (if applicable)

If the parent(s) of the subject or the investigator think that the subject doesn’t want to participate (anymore) in this research project the subject will leave the study.

11.4 Benefits and risks assessment, group relatedness

The burden and risks associated with participation to this study are minimal. The benefits of starting early complementary feeding are well described by King.[11] King suggested that it is not necessary to wait for lip seal to develop and tongue protrusion to diminish before

weaning. In fact, premature children may only mature with the aid of weaning. Furthermore, some infants may develop rapidly with increasingly textured food. There is evidence for not starting complementary feeding before 3 months corrected age, because motor development necessary for safe and successful transition to solid foods may not have been achieved until at least 3 months. For that reason, none of the children will start complementary feeding before 3 months of age corrected age. From data on term infants it seems likely that the later a preterm infant is introduced to new tastes, the less likely they are to accept a wide variety of foods. King also puts forward that there is no evidence that preterm infants are more likely to develop atopic diseases due to immaturity of the gut and immune system.

For the primary outcome we would like to determine BMI at the age of 2 years. Additionally, we would like to determine BMI before the start of complementary feeding and at the age of 1 year. Ideally, we would like to combine these hospital visits with regular visits in the follow-up of the preterm infants.
Furthermore, we will ask parents to collect faeces at home at three different timepoints. The faeces will be analysed to evaluate if the microbiota change due to the introduction of complementary feeding.

In conclusion, we would like to obtain information before the start of complementary feeding and at the age of 1 and 2 years. This means three extra hospital visits, depending on the arrangement for the follow-up of preterm infants in the different hospitals. Whenever possible, visits for the study will be combined with regular visits. The risks associated with this study are minimal. There will be no benefit for the individual participating in this study. To determine correlations between height, weight, BMI, and eating behaviour in preterm infants it is important to perform this study in this age category.

11.5 Compensation for injury

The sponsor/investigator has a liability insurance, which is in accordance with article 7, subsection 6 of the WMO.

The investigator also has an insurance which is in accordance with the legal requirements in the Netherlands (Article 7 WMO).This insurance provides cover for damage to research subjects through injury or death caused by the study (see section G1 of the research dossier).

The insurance applies to the damage that becomes apparent during the study or within 4 years after the end of the study.

11.6 Incentives (if applicable)

There will be no compensation through participation in the study.

12. ADMINISTRATIVE ASPECTS AND PUBLICATION

12.1 Handling and storage of data and documents

All data will be coded and will be therefore be as anonymously as possible. The identification code list can be used to link the data to the subject. This identification code list will be safeguarded by the investigators. Only the investigators will have access to the source data. The data will be stored for 15 years and the faeces samples will be stored for 10 years. Only mean and other statistical data of the individual data will be presented.

12.2 Monitoring and Quality Assurance

During this study, there is no use of a Data Safely Monitory Board. The risks associated with this study are minimal. The intervention of this study, namely the introduction of complementary feeding, will only be guided by giving advice of how to introduce solid foods.

12.3 Amendments

Amendments are changes made to the research after a favourable opinion by the accredited METC has been given. All amendments will be notified to the METC that gave a favourable opinion.

12.4 Annual progress report

The sponsor/investigator will submit a summary of the progress of the trial to the accredited METC once a year. Information will be provided on the date of inclusion of the first subject, numbers of subjects included and numbers of subjects that have completed the trial, serious adverse events/ serious adverse reactions, other problems, and amendments.

12.5 End of study report

The investigator will notify the accredited METC of the end of the study within a period of 8 weeks. The end of the study is defined as the last patient’s last visit.

In case the study is ended prematurely, the investigator will notify the accredited METC, including the reasons for the premature termination, within 15 days.

Within one year after the end of the study, the investigator/sponsor will submit a final

study report with the results of the study, including any publications/abstracts of the study, to the accredited METC.

12.6 Public disclosure and publication policy

Publication policy is according to the statement of the CCMO. Nor the sponsor, nor the investigators have veto in how publication of results will be. Results of this study will be submitted for publication, the investigators will initiate this.

Group 2a:

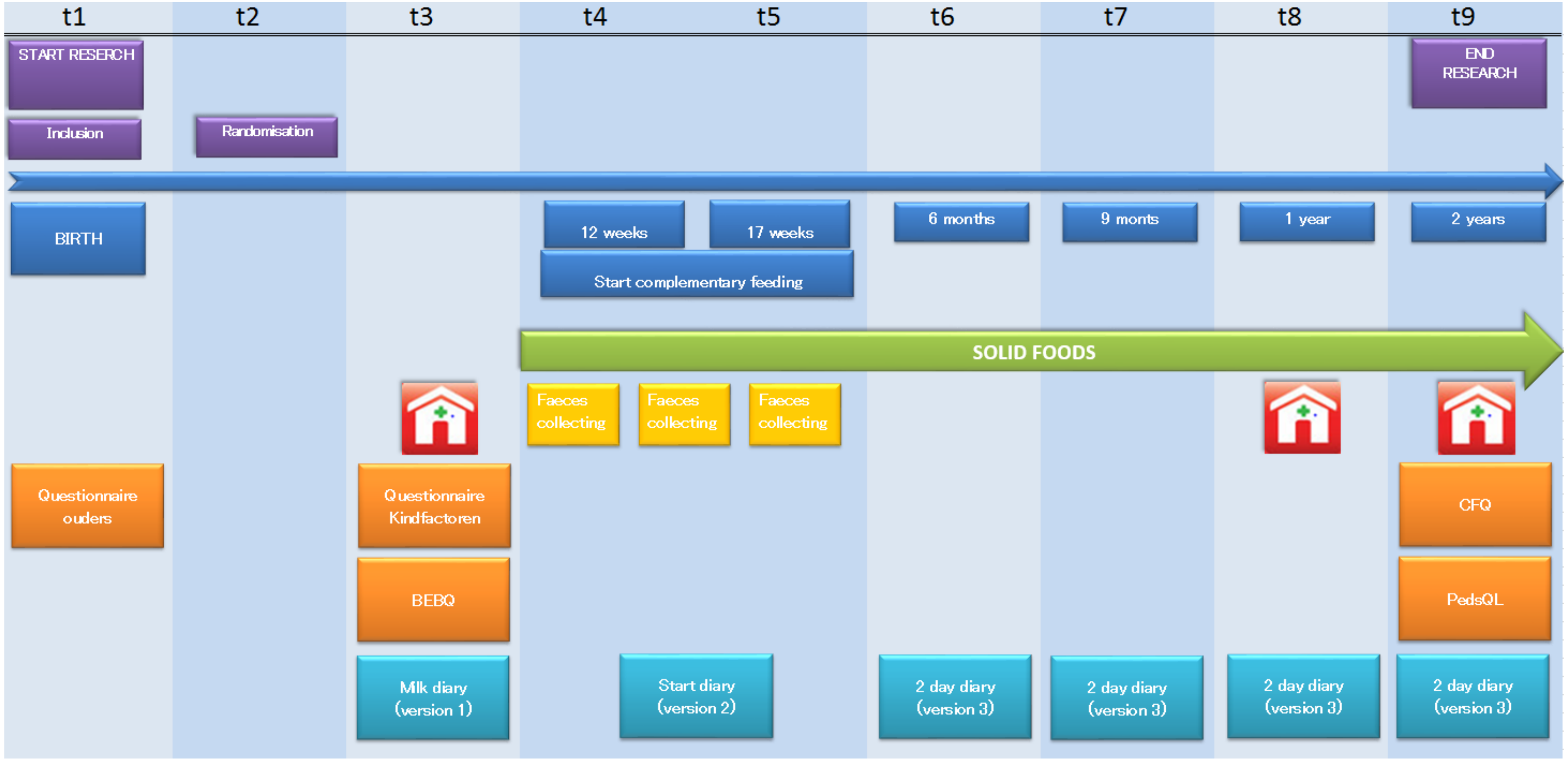
t1
t2
t3
t4
t5
t6
t7
t8
t9
START RESERCH
END RESEARCH
Inclusion
Randomisation
BIRTH
12 weeks
17 weeks
6 months
9 monts
1 year
2 years
Start complementary feeding
SOLID FOODS
Faeces collecting
Faeces collecting
Faeces collecting
Questionnaire ouders
Questionnaire Kindfactoren
CFQ
BEBQ
PedsQL
Milk diary (version 1)
Start diary (version 2)
2 day diary (version 3)
2 day diary (version 3)
2 day diary (version 3)
2 day diary (version 3)

Group 1:

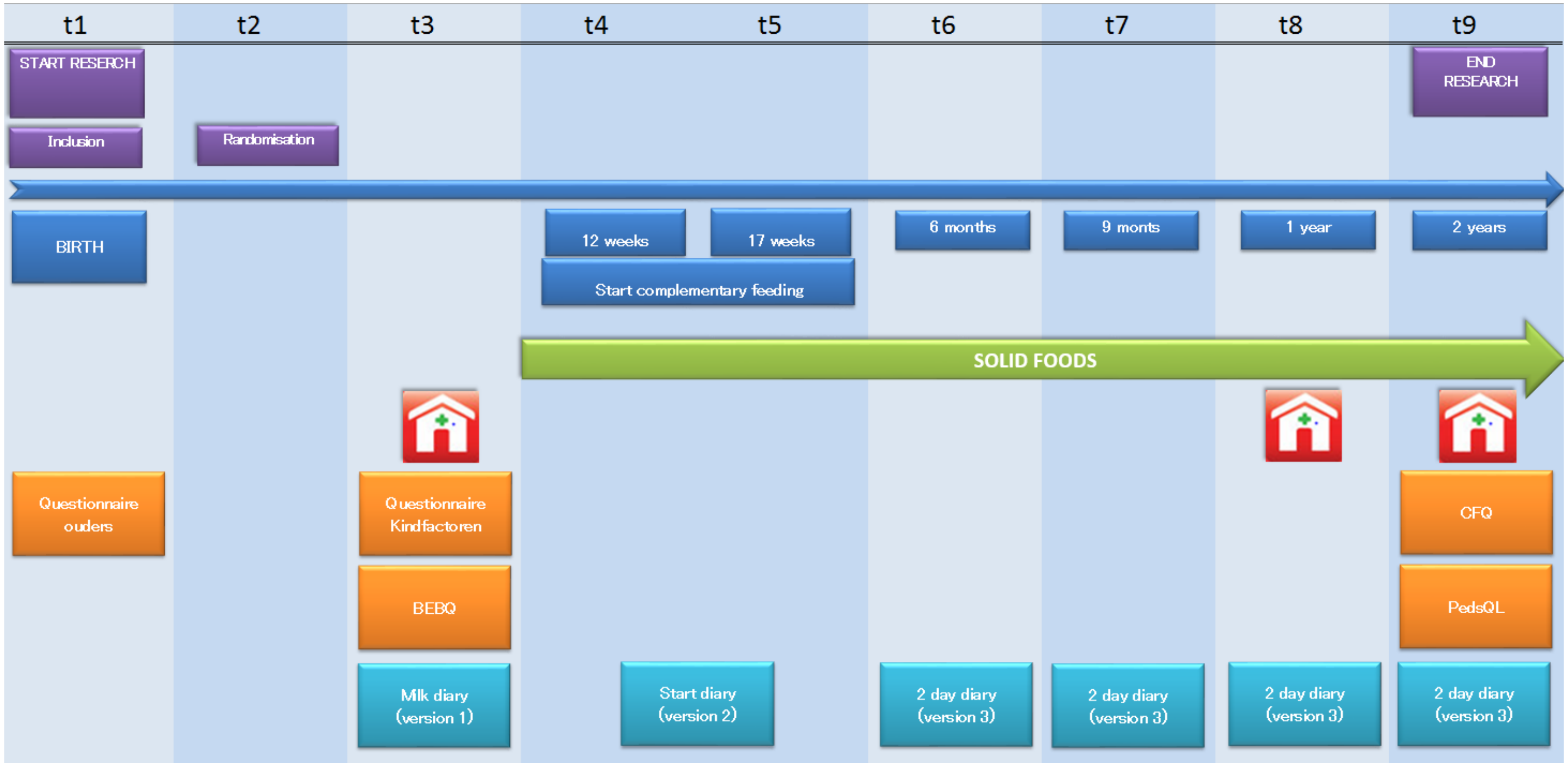
t1
t2
t3
t4
t5
t6
t7
t8
t9
START RESERCH
END RESEARCH
Inclusion
Randomisation
BIRTH
12 weeks
17 weeks
6 months
9 monts
1 year
2 years
Start complementary feeding
SOLID FOODS
Questionnaire ouders
Questionnaire Kindfactoren
BEBQ
CFQ
PedsQL
Milk diary (version 1)
Start diary (version 2)
2 day diary (version 3)
2 day diary (version 3)
2 day diary (version 3)
2 day diary (version 3)

Figure 1: Timeline

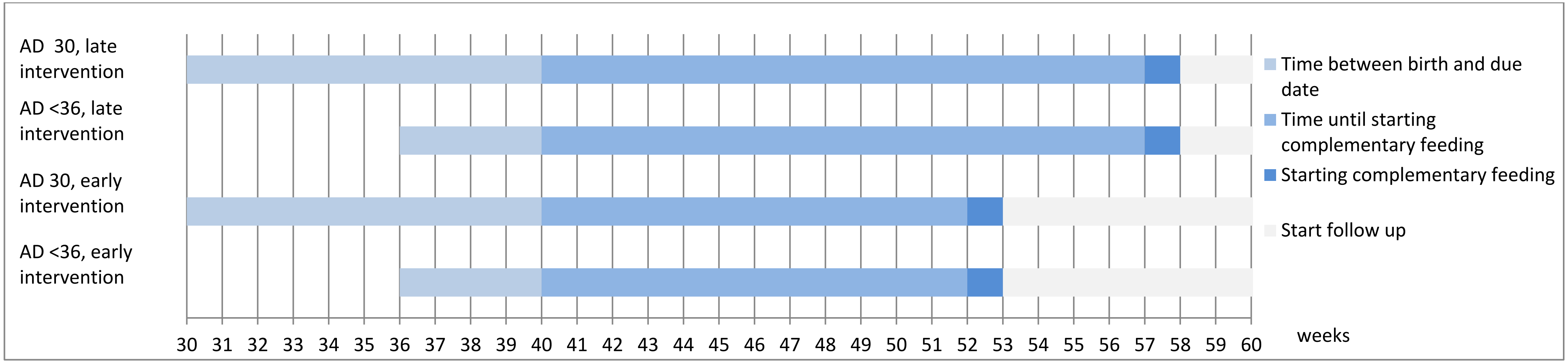

Figure 3: Showing the start of complementary feeding in the intervention and control group.

Reference List

(1) World Health Organization. Complementary feeding. Report of global consultation. Summary of guiding principles. 2002.
Ref Type: Online Source

(2) Agostoni C, Decsi T, Fewtrell M et al. Complementary feeding: a commentary by the ESPGHAN Committee on Nutrition. J Pediatr Gastroenterol Nutr 2008;46:99-110.

(3) Cattaneo A, Burmaz T, Arendt M et al. Protection, promotion and support of breast-feeding in Europe: progress from 2002 to 2007. Public Health Nutr 2010;13:751-759.

(4) World Health Organization. Global Strategy for Infant and Young Child Feeding. WHO [serial online] 2003.

(5) World Health Organization. The optimal duration of exclusive breastfeeding. Report of the expert consultation. WHO [serial online] 2001.

(6) World Health Organization. Complementary feeding: report of the global consultation. Summary of guiding principles. WHO [serial online] 2003.

(7) Cattaneo A, Williams C, Pallas-Alonso CR et al. ESPGHAN's 2008 recommendation for early introduction of complementary foods: how good is the evidence? Matern Child Nutr 2011;7:335-343.

(8) Schaaf JM, Mol BW, Abu-Hanna A, Ravelli AC. Trends in preterm birth: singleton and multiple pregnancies in the Netherlands, 2000-2007. BJOG 2011;118:1196-1204.

(9) Centraal bureau voor de statistiek. Statline. Bevolking en bevolkinsontwikkeling. http://statline cbs nl/StatWeb/publication [serial online] 2014.

(10) Palmer DJ, Makrides M. Introducing solid foods to preterm infants in developed countries. Ann Nutr Metab 2012;60 Suppl 2:31-8. doi: 10.1159/000335336. Epub;%2012 Apr 30.:31-38.

(11) King C. An evidence based guide to weaning preterm infants. Pediatrics and Child Health 2009;19:405-414.

(12) van Elburg RM, Fetter WP, Bunkers CM, Heymans HS. Intestinal permeability in relation to birth weight and gestational and postnatal age. Arch Dis Child Fetal Neonatal Ed 2003;88:F52-F55.

(13) Morgan JB, Lucas A, Fewtrell MS. Does weaning influence growth and health up to 18 months? Arch Dis Child 2004;89:728-733.

(14) D'Souza SW, Vale J, Sims DG, Chiswick ML. Feeding, growth, and biochemical studies in very low birthweight infants. Arch Dis Child 1985;60:215-218.

(15) Taylor TA, Kennedy KA. Randomized trial of iron supplementation versus routine iron intake in VLBW infants. Pediatrics 2013;131:e433-e438.

(16) Mills RJ, Davies MW. Enteral iron supplementation in preterm and low birth weight infants. Cochrane Database Syst Rev 2012;3:CD005095. doi: 10.1002/14651858.CD005095.pub2.:CD005095.

(17) *Baker RD, Greer FR. Diagnosis and prevention of iron deficiency and iron-deficiency anemia in infants and young children (0-3 years of age). Pediatrics 2010;126:1040-1050.*

(18) *Engelmann MD, Sandstrom B, Michaelsen KF. Meat intake and iron status in late infancy: an intervention study. J Pediatr Gastroenterol Nutr 1998;26:26-33.*

(19) *Jonsdottir OH, Thorsdottir I, Hibberd PL et al. Timing of the introduction of complementary foods in infancy: a randomized controlled trial. Pediatrics 2012;130:1038-1045.*

(20) *Marriott LD, Foote KD, Bishop JA, Kimber AC, Morgan JB. Weaning preterm infants: a randomised controlled trial. Arch Dis Child Fetal Neonatal Ed 2003;88:F302-F307.*

(21) *Morgan J, Williams P, Norris F, Williams CM, Larkin M, Hampton S. Eczema and early solid feeding in preterm infants. Arch Dis Child 2004;89:309-314.*

(22) *Eller E, Kjaer HF, Host A, Andersen KE, Bindslev-Jensen C. Food allergy and food sensitization in early childhood: results from the DARC cohort. Allergy 2009;64:1023-1029.*

(23) *Liem JJ, Kozyrskyj AL, Huq SI, Becker AB. The risk of developing food allergy in premature or low-birth-weight children. J Allergy Clin Immunol 2007;119:1203-1209.*

(24) *Kvenshagen B, Jacobsen M, Halvorsen R. Atopic dermatitis in premature and term children. Arch Dis Child 2009;94:202-205.*

(25) *Przyrembel H. Timing of introduction of complementary food: short- and long-term health consequences. Ann Nutr Metab 2012;60 Suppl 2:8-20.*

(26) *Pearce J, Taylor MA, Langley-Evans SC. Timing of the introduction of complementary feeding and risk of childhood obesity: a systematic review. Int J Obes (Lond) 2013;37:1295-1306.*

(27) *Jonsdottir OH, Kleinman RE, Wells JC et al. Exclusive breastfeeding for 4 versus 6 months and growth in early childhood. Acta Paediatr 2014;103:105-111.*

(28) *Seach KA, Dharmage SC, Lowe AJ, Dixon JB. Delayed introduction of solid feeding reduces child overweight and obesity at 10 years. Int J Obes (Lond) 2010;34:1475-1479.*

(29) *Huh SY, Rifas-Shiman SL, Taveras EM, Oken E, Gillman MW. Timing of solid food introduction and risk of obesity in preschool-aged children. Pediatrics 2011;127:e544-e551.*

(30) *Weng SF, Redsell SA, Swift JA, Yang M, Glazebrook CP. Systematic review and meta-analyses of risk factors for childhood overweight identifiable during infancy. Arch Dis Child 2012;97:1019-1026.*

(31) *Singh AS, Mulder C, Twisk JW, van MW, Chinapaw MJ. Tracking of childhood overweight into adulthood: a systematic review of the literature. Obes Rev 2008;9:474-488.*

(32) *Wahl S, Yu Z, Kleber M et al. Childhood obesity is associated with changes in the serum metabolite profile. Obes Facts 2012;5:660-670.*

(33) *Lira FS, Rosa JC, Dos Santos RV et al. Visceral fat decreased by long-term interdisciplinary lifestyle therapy correlated positively with interleukin-6 and tumor necrosis factor-alpha and negatively with adiponectin levels in obese adolescents. Metabolism 2011;60:359-365.*

(34) *Hamdy O, Porramatikul S, Al-Ozairi E. Metabolic obesity: the paradox between visceral and subcutaneous fat. Curr Diabetes Rev 2006;2:367-373.*

(35) *Giordano P, Del Vecchio GC, Cecinati V et al. Metabolic, inflammatory, endothelial and haemostatic markers in a group of Italian obese children and adolescents. Eur J Pediatr 2011;170:845-850.*

(36) *van Haastert IC, de Vries LS, Helders PJ, Jongmans MJ. Early gross motor development of preterm infants according to the Alberta Infant Motor Scale. J Pediatr 2006;149:617-622.*

(37) *HMSO. Department of health: weaning and the weaning diet. Report on Health and Social Subjects. 45. 1994. London. Ref Type: Report*

(38) *Yee J, Smith AM, O'Connor DL. Introduction of complementary foods in preterm infants varies among countries. J Am Diet Assoc 2001;101; A76:23.*

(39) *Morgan JB, Williams P, Foote KD, Marriott LD. Do mothers understand healthy eating principles for low-birth-weight infants? Public Health Nutr 2006;9:700-706.*

(40) *Norris FJ, Larkin MS, Williams CM, Hampton SM, Morgan JB. Factors affecting the introduction of complementary foods in the preterm infant. Eur J Clin Nutr 2002;56:448-454.*

(41) *Fanaro S, Borsari G, Vigi V. Complementary feeding practices in preterm infants: an observational study in a cohort of Italian infants. J Pediatr Gastroenterol Nutr 2007;45 Suppl 3:S210-4.:S210-S214.*

(42) *Cole TJ, Bellizzi MC, Flegal KM, Dietz WH. Establishing a standard definition for child overweight and obesity worldwide: international survey. BMJ 2000;320:1240-1243.*

(43) *Guideline on adjustment for baseline covariates. European medicines agency [serial online] 2013.*

(44) *D.G.Altman. Designing Research. Practical statistics for medical research. London: Chapman & Hall/CRC; 1999;74-103.*

(45) ***www.actiz.nl/jeugdgezondheidszorg/hmepage/feiten-en-cijfers**. 2014.*
Ref Type: Internet Communication

(46) *Nederlands Centrum Jeugdgezondheidszorg. JGZ-richtlijn Contactmomenten basistakenpakket Jeugdgezondheidszorg 0-19 jaar. 2014.*
Ref Type: Report

(47) *Jobe AH, Bancalari E. Bronchopulmonary dysplasia. Am J Respir Crit Care Med 2001;163:1723-1729.*

(48) *Jingxiong J, Rosenqvist U, Huishan W et al. Relationship of parental characteristics and feeding practices to overweight in infants and young children in Beijing, China. Public Health Nutr 2009;12:973-978.*

(49) *Young BE, Johnson SL, Krebs NF. Biological determinants linking infant weight gain and child obesity: current knowledge and future directions. Adv Nutr 2012;3:675-686.*

(50) *Dubois L, Girard M. Early determinants of overweight at 4.5 years in a population-based longitudinal study. Int J Obes (Lond) 2006;30:610-617.*

(51) *Schonbeck Y, Talma H, van DP et al. Increase in prevalence of overweight in Dutch children and adolescents: a comparison of nationwide growth studies in 1980, 1997 and 2009. PLoS One 2011;6:e27608.*

(52) *Bocca-Tjeertes IF, Kerstjens JM, Reijneveld SA, de Winter AF, Bos AF. Growth and predictors of growth restraint in moderately preterm children aged 0 to 4 years. Pediatrics 2011;128:e1187-e1194.*

(53) *Euser AM, de Wit CC, Finken MJ, Rijken M, Wit JM. Growth of preterm born children. Horm Res 2008;70:319-328.*

(54) *Severity scoring of atopic dermatitis: the SCORAD index. Consensus Report of the European Task Force on Atopic Dermatitis. Dermatology 1993;186:23-31.*

(55) *Eiser C, Jenney M. Measuring quality of life. Arch Dis Child 2007;92:348-350.*

(56) *Raat H, Landgraf JM, Oostenbrink R, Moll HA, Essink-Bot ML. Reliability and validity of the Infant and Toddler Quality of Life Questionnaire (ITQOL) in a general population and respiratory disease sample. Qual Life Res 2007;16:445-460.*

(57) *Oostenbrink R, Jansingh-Piepers EM, Raat H et al. Health-related quality of life of pre-school children with wheezing illness. Pediatr Pulmonol 2006;41:993-1000.*

(58) *Oostenbrink R, Spong K, de Goede-Bolder A, Landgraf JM, Raat H, Moll HA. Parental reports of health-related quality of life in young children with neurofibromatosis type 1: influence of condition specific determinants. J Pediatr 2007;151:182-6, 186.*

(59) *Oostenbrink R, Jongman H, Landgraf JM, Raat H, Moll HA. Functional abdominal complaints in pre-school children: parental reports of health-related quality of life. Qual Life Res 2010;19:363-369.*

(60) *Squire J TEBDPL. ASQ-3: User's guide. 2016. Baltimore: Paul H. Brookes Publishing Co.; 2009.*

(61) *Kyle UG, Bosaeus I, De Lorenzo AD et al. Bioelectrical impedance analysis-part II: utilization in clinical practice. Clin Nutr 2004;23:1430-1453.*

(62) *Kyle UG, Bosaeus I, De Lorenzo AD et al. Bioelectrical impedance analysis--part I: review of principles and methods. Clin Nutr 2004;23:1226-1243.*

(63) *Urlando A, Dempster P, Aitkens S. A new air displacement plethysmograph for the measurement of body composition in infants. Pediatr Res 2003;53:486-492.*

(64) *Sainz RD, Urlando A. Evaluation of a new pediatric air-displacement plethysmograph for body-composition assessment by means of chemical analysis of bovine tissue phantoms. Am J Clin Nutr 2003;77:364-370.*

(65) *Dempster P, Aitkens S. A new air displacement method for the determination of human body composition. Med Sci Sports Exerc 1995;27:1692-1697.*

(66) Broder-Fingert S, Crowley WF, Jr., Boepple PA. Safety of frequent venous blood sampling in a pediatric research population. J Pediatr 2009;154:578-581.

(67) World Medical Association Declaration of Helsinki: ethical principles for medical research involving human subjects. JAMA 2013;310:2191-2194.
