# Supplementary material for: Timing of Complementary Feeding in Preterm Infants and Prevalence of Overweight and Obesity: A Randomized Clinical Trial
Source: JAMA Netw Open. 2025 Apr 30;8(4):e252968. doi: 10.1001/jamanetworkopen.2025.2968 (PMC12044495; doi:10.1001/jamanetworkopen.2025.2968)
Supplement: Supplement 2. — eTable 1. Characteristics of the Study Population Before Randomization (for Preterm Infants) or at 3 Months (for Full-Term Infants) eTable 2. Growth Parameters of Preterm Infants at Corrected Age 1 Year, Including Mixed Model With Correction for Correlations eTable 3. Health-Related Quality of Life: PedsQL Infant Scales eTable 4. Neurodevelopment: ASQ-3 at Correct Age 1 Year and 2 Years for Preterm Infants eTable 5. SCORAD for Atopic Dermatitis in Preterm Infants eTable 6. Initiating Complementary Feeding eTable 7. Per-Protocol Analysis: Growth Parameter of Preterm Infants at Correct Age 2 Years, Including Mixed model for Repeated Measurements eFigure. Change in Growth Parameters Over Time Until (Corrected) Age 2 Years for Preterm and Full-Term Infants eReference. [file jamanetwopen-e252968-s002.pdf]

Supplemental Online Content

Vissers KM, Feskens EJM, van Goudoever JB, Janse AJ; SPOON Study Group. Timing of complementary feeding in preterm infants and prevalence of overweight and obesity: a randomized clinical trial. *JAMA Netw Open*. 2025;8(4):e252968. doi:10.1001/jamanetworkopen.2025.2968

eTable 1. Characteristics of the Study Population Before Randomization (for Preterm Infants) or at 3 Months (for Full-Term Infants)
eTable 2. Growth Parameters of Preterm Infants at Corrected Age 1 Year, Including Mixed Model With Correction for Correlations
eTable 3. Health-Related Quality of Life: PedsQL Infant Scales
eTable 4. Neurodevelopment: ASQ-3 at Correct Age 1 Year and 2 Years for Preterm Infants
eTable 5. SCORAD for Atopic Dermatitis in Preterm Infants
eTable 6. Initiating Complementary Feeding
eTable 7. Per-Protocol Analysis: Growth Parameter of Preterm Infants at Correct Age 2 Years, Including Mixed model for Repeated Measurements
eFigure. Change in Growth Parameters Over Time Until (Corrected) Age 2 Years for Preterm and Full-Term Infants
eReference

This supplemental material has been provided by the authors to give readers additional information about their work.

© 2025 Vissers KM et al. *JAMA Network Open*.

eTable 1. Characteristics of the Study Population Before Randomization (for Preterm Infants) or at 3 Months (for Full-Term Infants)

		**Early group preterm infants**	**Late group preterm infants**	**Term infants**
Corrected age (weeks) at measurements	mean (sd)	10.60 (1.76)	10.85 (2.13)	12.2 (1.79)
Height in cm	mean (sd)	59.03 (2.32)	59.03 (2.90)	61.13 (2.41)
Height-for-age z-score	mean (sd)	-0.19 (1.03)	-0.24 (1.05)	0.40 (0.89)
Weight in grams	mean (sd)	5511 (730)	5467 (833)	6061 (818)
Weight-for-age z-score	mean (sd)	-0.45 (1.05)	-0.48 (0.96)	0.018 (0.90)
Weight-for-height z-score	mean (sd)	-0.37 (0.97)	-0.33 (0.97)	-0.28 (0.94)
Head circumference in cm	mean (sd)	40.02 (1.42)	40.07 (1.61)	40.67 (1.32)
Head circumference-for-age z-score	mean (sd)	0.45 (0.56)	0.52 (1.07)	0.68 (0.85)
Breast milk feeding / Formula feeding / Combination	n (%)	28 (21.2) / 79 (59.8) / 17 (12.9)	23 (18.3) / 76 (60.3) / 21 (16.7)	75 (47.2) / 61 (38.4) / 20 (12.6)
Iron suppletion	Yes/No	43 (32.6) / 81 (61.4)	39 (31.0) / 82 (65.1)	0 /159 (100)
**BEBQ score**[a]				
Enjoyment of Food [b]	mean (sd)	4.2 (0.44)	4.2 (0.47)	4.2 (0.45)
Food Responsiveness [c]	mean (sd)	2.3 (0.62)	2.2 (0.68)	2.2 (0.68)
Slowness in Eating [b]	mean (sd)	2.5 (0.72)	2.5 (0.71)	2.4 (0.76)
Satiety Responisveness [d]	mean (sd)	2.5 (0.69)	2.5 (0.79)	2.4 (0.68)
General Appetite [e]	mean (sd)	3.5 (0.80)	3.4 (1.02)	3.6 (0.86)

a BEBQ: Likert response scale of 1 to 5 (1 = never, 2 = rarely, 3 = sometimes, 4 = often, 5 = always). BEBQ contains 18 items in total (b=4 items; c=6 items, d=3 items, e=1 item)

© 2025 Vissers KM et al. *JAMA Network Open*.

eTable 2. Growth Parameters of Preterm Infants at Corrected Age 1 Year, Including Mixed Model With Correction for Correlations

	**Early group preterm infants**	**Late group preterm infants**			**Model 1[m]**		**Term infants**
	mean (sd)	*mean (sd)*	*Between-group mean difference (95%CI)*	*p-value*	*Regression coefficient (95% CI)*	*p-value*	*mean (sd)*
Height (cm)	75.44 (2.79)[a]	75.97 (2.80)[d]	-0.53 (-1.2 to 0.19)	0.15	1.39 (-2.69 to 5.47)	0.50	75.40 (2.71)[g]
Height-for-age z-score	0.11 (1.04)[a]	0.29 (1.07)[d]	-0.18 (-0.45 to 0.09)	0.19	-0.18 (0.45 to0.09)	0.19	0.54 (1.07)[g]
Weight (kilogram)	9.52 (1.08) [b]	9.64 (1.14)[e]	-0.12 (-0.41 to 0.16)	0.40	-0.12 (-0,40 to 0,15)	0.38	9.78 (1.08)[g]
Weight-for-age z-score	0.07 (0.98)[b]	0.21 (0.97)[e]	-0.14 (-0.39 to 0.11)	0.27	-0.14 (-0.39 to 0.11)	0.26	0.55 (0.90)[g]
Weight-for-height z-score	0.04 (1.01)[a]	0.16 (0.91)[e]	-0.12 (-0.37 to 0.13)	0.33	-0.12 (-0.37 to 0.12)	0.33	0.42 (0.89)[g]
Head circumference (cm)	46.14 (1.54)[c]	46.41 (1.64)[f]	-0.27 (-0.74 to 0.21)	0.27	11.14 (-3.04 to 25.32)	0.12	46.40 (1.35)[h]
Head circumference-for-age z-score	0.45 (1.06)[c]	0.66 (1.16)[f]	-0.21 (-0.54 to 0.12)	0.22	-0.21 (-0.54 to 0.12)	0.21	0.91 (0.94)[h]
	n (%)	*n (%)*		*p-value*	*OR (95% CI)*		*n (%)*
Normal weight / overweight & obesity according to WHO definition	120 (99.2) / 1 (0.8)[a]	112 (98.2) / 2 (1.8)[e]		0.53	0.47 (0.04 - 5.24)	0.54	146 (95.4) / 7 (4.6)[g]

Amount of patients with complete data a: n=121; b: n=122; c: n=95; d: n=116; e: n=114; f: n=79; g: n=153; h: n=145
m = Model 1: Mixed model for repeated measurements with correction for ID, hospital site and twins

eTable 3. Health-Related Quality of Life: PedsQL Infant Scales[a]

		Early group preterm infants	Late group preterm infants	p-value
Physical Functioning [b]	Median (IQR)	83.33 (74.31-88.89)	83.33 (75.00-91.67)	0.77
Physical Symptoms [c]	Median (IQR)	87.26 (82.50-95.00)	87.50 (80.00-95.00)	0.05
Emotional Functioning [d]	Median (IQR)	72.92 (64.58-81.25)	72.92 (64.58-81.25)	0.77
Social Functioning [e]	Median (IQR)	92.50 (80.00-95.00)	90.00 (85.00-100.0)	0.26
Cognitive Functioning [f]	Median (IQR)	80.56 (69.45-88.89)	77.78 (69.44-94.44)	0.33
Psychosocial Health Summary Score	Median (IQR)	77.88 (70.19-84.62)	78.85 (71.15-85.58)	0.70
Physical Health Summary Score	Median (IQR)	86.84 (81.58-90.79)	85.53 (75.00-92.11)	0.46
Total score	Median (IQR)	80.83 (75.56-86.67)	81.67 (72.22-87.22)	0.99

a PedsQL: Likert response scale of 1 to 5 (1 = never, 2 = almost never, 3 = sometimes, 4 = often, 5 = almost always). PedsQL contains 45 items in total (b=9 items; c=10 items, d=12 items, e=5 item, f=9 items)

© 2025 Vissers KM et al. *JAMA Network Open*.

eTable 4. Neurodevelopment: ASQ-3 at Correct Age 1 Year and 2 Years for Preterm Infants[a]

**ASQ 1 year**		**Early group preterm infants**	**Late group preterm infants**	**p-value**
Communication (normal / -1SD / -2SD)	n (%)	100 (85.5) / 15 (12.8) / 2 (1.7)	89 (80.9) / 17 (15.5) / 4 (3.6)	0.54
Gross motor (normal / -1SD / -2SD)	n (%)	97 (82.9) / 20 (17.1) / 0 (0)	91 (82.7) / 19 (17.3) / 0 (0)	0.97
Fine motor (normal / -1SD / -2SD)	n (%)	99 (84.6) / 11 (9.4) / 7 (6.0)	94 (85.5) / 7 (6.4) / 9 (8.2)	0.59
Problem solving (normal / -1SD / -2SD)	n (%)	105 (89.7) / 9 (7.7) / 3 (2.6)	94 (85.5) / 11 (10.0) / 5 (4.5)	0.58
Personal-social (normal / -1SD / -2SD)	n (%)	94 (81.0) / 18 (15.5) / 4 (3.4)	85 (78.0) / 21 (19.3) / 3 (2.8)	0.74
Total score (normal / -1SD / -2SD)	n (%)	100 (86.2) / 12 (10.3) / 4 (3.4)	95 (87.2) / 10 (9.2) / 4 (3.7)	0.96
Total score below 1SD	n (%)	16 (12.2)	14 (11.3)	0.83
Mean score in one domain below 2SD	n (%)	10 (7.6)	14 (11.3)	0.31
**ASQ 2 years**		**Early group preterm infants**	**Late group preterm infants**	**p-value**
Communication (normal / -1SD / -2SD)	n (%)	92 (80.7) / 14 (12.3) / 8 (7.0)	85 (84.2) / 9 (8.9) / 7 (6.9)	0.72
Gross motor (normal / -1SD / -2SD)	n (%)	85 (74.6) / 10 (8.8) / 19 (16.7)	77 (75.5) / 15 (14.7) / 10 (9.8)	0.17
Fine motor (normal / -1SD / -2SD)	n (%)	96 (84.2) / 6 (5.3) / 12 (10.5)	79 (78.2) / 11 (10.9) / 11 (10.9)	0.30
Problem solving (normal / -1SD / -2SD)	n (%)	87 (77.0) / 21 (18.6) / 5 (4.4)	75 (74.3) / 17 (16.8) / 9 (8.9)	0.41
Personal-social (normal / -1SD / -2SD)	n (%)	95 (84.8) / 11 (9.8) / 6 (5.4)	91 (89.2) / 4 (3.9) / 7 (6.9)	0.23
Total score (normal / -1SD / -2SD)	n (%)	90 (80.4) / 11 (9.8) / 11 (9.8)	81 (80.2) / 12 (11.9) / 8 (7.9)	0.81
Total score below 1SD	n (%)	22 (16.8)	20 (16.1)	0.98
Mean score in one domain below 2SD	n (%)	30 (22.9)	25 (20.2)	0.71

a ASQ-3: The ASQ-3 concerns five developmental domains: Communication, Gross Motor, Fine Motor, Problem Solving and Personal Social. Each item is indicated as “yes”, “sometimes”, or “not yet”. SD-scores based on Steenis et al. (1)

© 2025 Vissers KM et al. *JAMA Network Open*.

eTable 5. SCORAD for Atopic Dermatitis in Preterm Infants

		**Early group preterm infants**	**Late group preterm infants**	**p-value**
SCORAD at randomization<br>(mild/moderate/severe)	<25 / 25-50 / >50	97 (98.0) / 2 (2.0) / 0 (0)	96 (100) / 0 (0) / 0 (0)	0.16
SCORAD at 1 year of age<br>(mild/moderate/severe)	<25 / 25-50 / >50	90 (93.8) / 6 (6.3) / 0 (0)	93 (100) / 0 (0) / 0 (0)	0.01
SCORAD at 2 years of age<br>(mild/moderate/severe)	<25 / 25-50 / >50	72 (93.5) / 5 (6.5) / 0 (0)	58 (90.6) / 4 (6.3) / 2 (3.1)	0.30

© 2025 Vissers KM et al. *JAMA Network Open*.

eTable 6. Initiating Complementary Feeding

		Early group preterm infants	Late group preterm infants	p-value	Term infants
Type of complementary feeding starting with	Homemade / Commercial (n (%))	106 (86.2) / 17 (13.8)	90 (76.9) / 27 (23.1)	0.06	122 (79.2) / 32 (20.8)
Kind of complementary feeding starting with	Vegetables / Other (n (%))[a]	114 (92.7) / 9 (7.3)	91 (77.8) / 26 (22.2)	0.001	106 (68.8) / 48 (31.2)

a = fruit, oats, bread, warm meal

© 2025 Vissers KM et al. *JAMA Network Open*.

eTable 7. Per-Protocol Analysis: Growth Parameter of Preterm Infants at Correct Age 2 Years, Including Mixed model for Repeated Measurements

	**Early group preterm infants**	**Late group preterm infants**			**Model 1[m]**	
	mean (sd)	*mean (sd)*	*Between-group mean difference (95%CI)*	*p-value*	*Regression coefficient (95% CI)*	*p-value*
Height (cm)	87.39 (3.64)[a]	87.60 (3.71)[d]	-0.21 (-1.22 to 0.80)	0.68	1.95 (-1.58 to 5.48)	0.28
Height-for-age z-score	0.16 (1.19)[a]	0.19 (1.13)[d]	-0.03 (-0.36 to 0.29)	0.85	-0.11 (-3.54 to 0.14)	0.38
Weight (kg)	12.44 (1.47)[b]	12.53 (1.57)[d]	-0.10 (-0.51 to 0.32)	0.65	-0.09 (-0.57 to 0.39)	0.71
Weight-for-age z-score	0.32 (1.07)[b]	0.39 (1.01)[d]	-0.07 (-0.36 to 0.22)	0.64	-0.13 (-0.36 to 0.11)	0.29
Weight-for-height z-score	0.27 (1.09)[a]	0.35 (0.90)[d]	-0.08 (-0.36 to 0.20)	0.57	-0.13 (-0.35 to 0.08)	0.23
Head circumference (cm)	49.13 (1.48)[c]	48.63 (1.95)[e]	0.50 (-0.19 to 1.20)	0.16	-0.23 (-9.46 to 9.01)	0.96
Head circumference-for-age z-score	0.91 (1.06)[c]	0.69 (1.30)[e]	0.22 (-0.26 to 0.70)	0.36	-0.09 (-0.35 to 0.18)	0.52
BMI (kg/m2)	16.25 (1.45)[a]	16.28 (1.25)[d]	-0.03 (-0.40 to 0.35)	0.88	-0.12 (-0.42 to 0.19)	0.46
BMI-for-age z-score	0.31 (1.13)[a]	0.39 (0.91)[d]	-0.08 (-0.36 to 0.21)	0.59	-0.12 (-0.34 to 0.10)	0.29
	n (%)	*n (%)*			*OR (95% CI)*	*p-value*
Normal / overweight & obesity according to IOTF definition	101 (92.7) / 8 (7.3)	90 (93.8) / 6 (6.3)		0.76	1.14 (0.37 - 3.53)	0.83
Normal / overweight & obesity according to WHO definition	106 (97.2) / 3 (2.8)	92 (95.8) / 4 (4.2)		0.58	0.58 (0.12 - 2.91)	0.51

Amount of patients with complete data a: n=109; b: n=111; c: n=54; d: n=96; e: n=42
m = Model 1: Mixed model for repeated measurements with correction for ID, hospital site and twins

© 2025 Vissers KM et al. *JAMA Network Open*.

eFigure. Change in Growth Parameters Over Time Until (Corrected) Age 2 Years for Preterm and Full-Term Infants

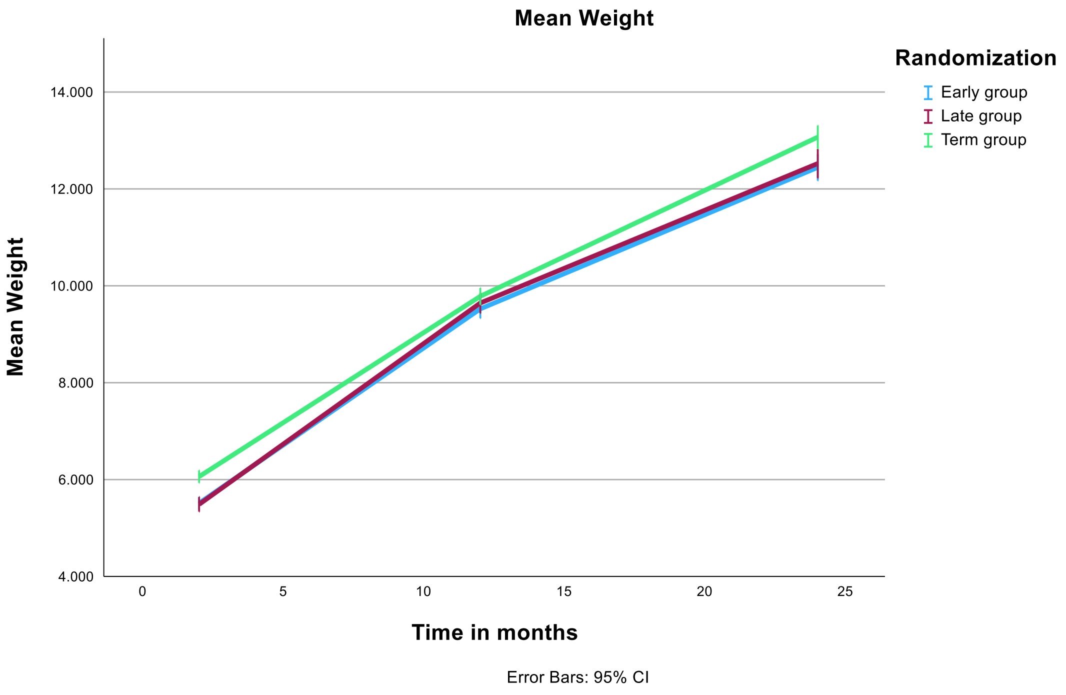

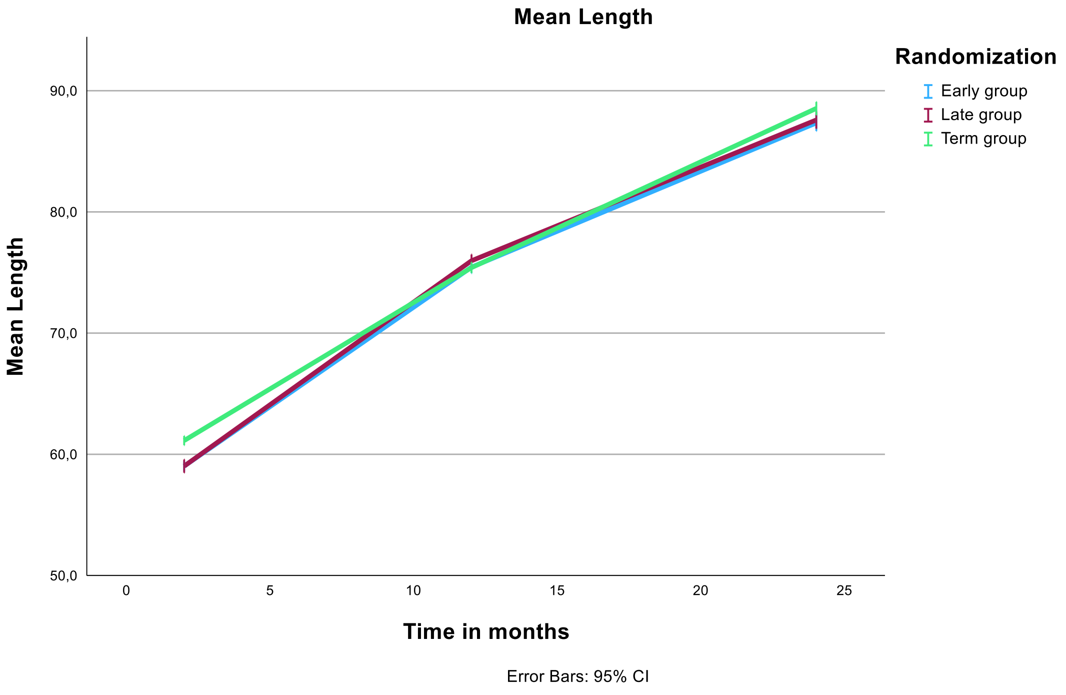

© 2025 Vissers KM et al. *JAMA Network Open*.

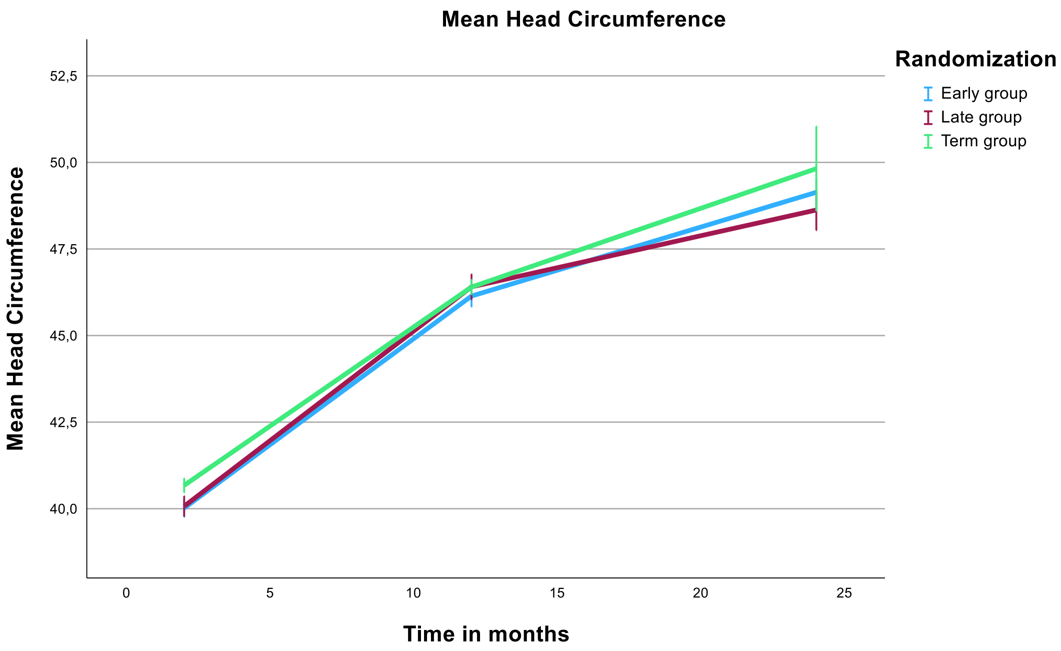

Error Bars: 95% CI

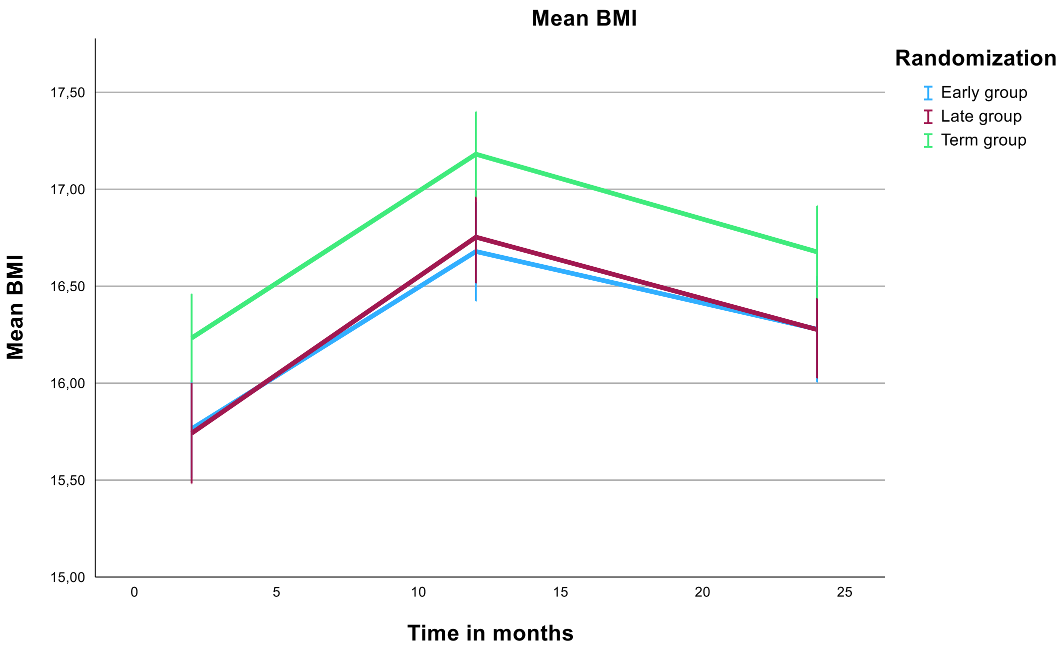

Error Bars: 95% CI

© 2025 Vissers KM et al. *JAMA Network Open*.

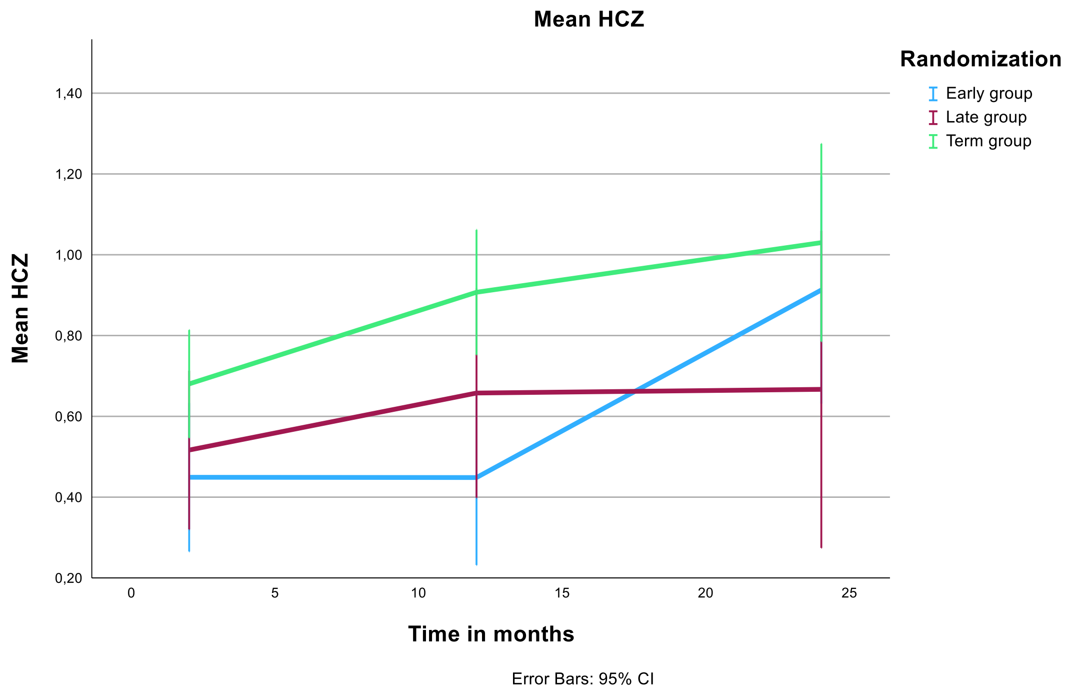

All data: mean (sd). Time in months is corrected for age for preterm infants. HCZ = Head Circumference-for-age z-score.

eReference

1. Steenis LJP, Verhoeven M, Hessen DJ, van Baar AL. Parental and professional assessment of early child development: The ASQ-3 and the Bayley-III-NL. *Early Hum Dev*. 2015;91(3):217-225. doi:10.1016/j.earlhumdev.2015.01.008

© 2025 Vissers KM et al. *JAMA Network Open*.
