# Supplementary material for: Timing of Complementary Feeding in Preterm Infants and Prevalence of Overweight and Obesity: A Randomized Clinical Trial
Source: JAMA Netw Open. 2025 Apr 30;8(4):e252968. doi: 10.1001/jamanetworkopen.2025.2968 (PMC12044495; doi:10.1001/jamanetworkopen.2025.2968)
Supplement: Supplement 3. — Nonauthor Collaborators [file jamanetwopen-e252968-s003.pdf]

Supplemental Online Content: Nonauthor Collaborators
*First name, last name, and suffix (if applicable) are required and will appear in PubMed.

| *Group Name(s): SPOON-study group | | | | | | | |
|---|---|---|---|---|---|---|---|
| ***First Name and Middle Initial(s)** | ***Last Name** | ***Suffix (eg, Jr, III)** | Academic Degrees | Institution | Location (city, state/province, country) | Role or Contribution, eg, chair, principal investigator | Group (if more than 1 Group listed in the byline) and/or Subgroup (eg, Steering Committee) |
| Martin G.A. | Baartmans | | MD, PhD | Maasstad Hospital, Department of Pa | Rotterdam, The Netherlands | local coordinating investigator | SPOON-study group |
| Ron H.T. | van Beek | | MD, PhD | Amphia Hospital, Department of Paed | Breda, The Netherlands | local coordinating investigator | SPOON-study group |
| Frans B. | Plotz | | MD, PhD | Ter Gooi Medical Center, Department of Paediatrics / Amsterdam UMC, location University of Amsterdam, Emma Children's Hospital, Department of Paediatrics and Amsterdam Reproduction & Development Research Institute | Blaricum, the Netherlands / Amsterdam, the Netherlands | local coordinating investigator | SPOON-study group |
| Marianne | Eijkermans | | MD, PhD | Catharina Hospital, Department of Paediatrics | Eindhoven, the Netherlands | local coordinating investigator | SPOON-study group |
| Angelique K.E. | Hoffmann-Haringsma | | MD | Franciscus Gasthuis en Vlietland Hosp | Rotterdam, The Netherland | local coordinating investigator | SPOON-study group |
| Jeroen H.L | van Hoorn | | MD | Viecuri Medical Centrum, Departmen | Venlo, The Netherlands | local coordinating investigator | SPOON-study group |
| Christine H. | ten Hove | | MD | Rijnstate Hospital, Department of Pae | Arnhem, The Netherlands | local coordinating investigator | SPOON-study group |
| Monique A.M. | Jacobs | | MD | ingeland Hospital, Department of Pae | Doetinchem, The Netherla | local coordinating investigator | SPOON-study group |
| Jorien M. | Kerstjens | | MD, PhD | Beatrix Children's Hospital, University | Groningen, The Netherlands | local coordinating investigator | SPOON-study group |
| Henriette | van Laerhoven | | MD | OLVG Amsterdam, Department of Pe | Amsterdam, The Netherlan | local coordinating investigator | SPOON-study group |
| Jeannette S. | von Lindern | | MD, PhD | Groene Hart Hospital, Department of Paediatrics | Gouda, The Netherlands | local coordinating investigator | SPOON-study group |

Supplemental Online Content: Nonauthor Collaborators
*First name, last name, and suffix (if applicable) are required and will appear in PubMed.

| ***First Name and Middle Initial(s)** | ***Last Name** | ***Suffix (eg, Jr, III)** | Academic Degrees | Institution | Location (city, state/province, country) | Role or Contribution, eg, chair, principal investigator | Group (if more than 1 Group listed in the byline) and/or Subgroup (eg, Steering Committee) |
|---|---|---|---|---|---|---|---|
| Dianne A.P.G.F. | Maingay-Visser | | MD | DeKinderkliniek, Department of Paediatrics | Almere, The Netherlands | local coordinating investigator | SPOON-study group |
| Clemens B. | Meijssen | | MD | Meander Medical Centre, Department of Paediatrics | Heerlen, The Netherlands | local coordinating investigator | SPOON-study group |
| Rob M.J. | Moonen | | MD, PhD | Zuyderland Medical Centre, Department of Paediatrics | Amersfoort, The Netherlan | local coordinating investigator | SPOON-study group |
| Annemarie J.H. | Oudshoorn | | MD, PhD | Gelre Hospital, Department of Paedia | Apeldoorn, The Netherlands | local coordinating investigator | SPOON-study group |
| Linda G.M. | van Rooij | | MD | Medisch Spectrum Twente, Department of Paediatrics | Enschede, The Netherlands | local coordinating investigator | SPOON-study group |
| Sophie R.D. | van der Schoor | | MD, PhD | OLVG Amsterdam, Department of Pediatrics | Amsterdam, The Netherlands | local coordinating investigator | SPOON-study group |
