# Supplementary material for: Timing of Complementary Feeding in Preterm Infants and Prevalence of Overweight and Obesity: A Randomized Clinical Trial
Source: JAMA Netw Open. 2025 Apr 30;8(4):e252968. doi: 10.1001/jamanetworkopen.2025.2968 (PMC12044495; doi:10.1001/jamanetworkopen.2025.2968)
Supplement: Supplement 4. — Data Sharing Statement [file jamanetwopen-e252968-s004.pdf]

# Data Sharing Statement

Vissers. Timing of Complementary Feeding in Preterm Infants and Prevalence of Overweight and Obesity. *JAMA Netw Open*. Published April 30, 2025.
doi:10.1001/jamanetworkopen.2025.2968

**Data**
**Additional Information:** https://onderzoekmetmensen.nl/en/trial/53076
**Data available:** Yes
**Data types:** Deidentified participant data, Other (please specify)
**Additional Information:** On request
**How to access data:** On request: janseA1@zgv.nl
**When available:** With publication

**Supporting Documents**
**Document types:** None

**Additional Information**
**Who can access the data:** researchers whose proposed use of the data has been approved
**Types of analyses:** any purpose
**Mechanisms of data availability:** signed data access agreement
